# How can we compare multispecies livestock rearing households? – an analysis of the impact of health and production parameters on multispecies livestock rearing outcomes

**DOI:** 10.1186/s12917-022-03175-x

**Published:** 2022-04-29

**Authors:** Tu Tu Zaw Win, Angus Campbell, Ricardo J. Soares Magalhaes, Kyaw Naing Oo, Joerg Henning

**Affiliations:** 1 The School of Veterinary Science, The University of Queensland, Gatton, Australia; 2 Faculty of Veterinary & Agricultural Sciences, The University of Melbourne, Melbourne, Australia; 3 UQ Spatial Epidemiology Laboratory, The School of Veterinary Science, The University of Queensland, Gatton, Australia; 4 Children’s Health and Environment Program, The University of Queensland, The University of Queensland, QLD, South Brisbane, 4101 Australia; 5 Livestock Breeding and Veterinary Department, The Ministry of Agriculture, Livestock and Irrigation, Naypyidaw, Myanmar; 6 The School of Veterinary and Life Sciences, College of Veterinary Medicine, Murdoch University, Perth, 6150 Western Australia

**Keywords:** Multispecies, Syndromic health problems, Biosecurity, Income

## Abstract

**Background:**

The Central Dry Zone (CDZ) of Myanmar is a critical region of livestock production. This region supports 10 million people whose livelihoods depend on small-scale, dry-land agriculture, but it is also one of the poorest regions of Myanmar. Little is known about the constraints to animal health in multi-species livestock farms in this region or the relationships between husbandry practices and measures of the success of livestock rearing such as income, and successful health management.

**Results:**

In this study, we describe associations between husbandry practices and animal health problems affecting different body systems. We also develop a biosecurity and livestock disease prevention index by taking account of different activities (i.e. treatment, vaccination, reducing disease transmission practice, sanitation) that can be compared between livestock species, estimate the income generated from livestock production, and identify factors influencing these parameters. Cross-sectional study was used to collect data on livestock production and health from cattle (*N* = 382), sheep, goat (*N* = 303) and village chicken (*N* = 327) farmers in 40 villages of the CDZ. Survey-design based techniques and F-statistics, ordinal, and binomial regression were used for data analysis. Our results indicate that a significant proportion of farmers’ income in the CDZ comes from crop production (43.2%) and livestock production (23.1%) and the rest of the farmers’ income is derived from trading, supported by other relatives and employment. Our results indicate that animal health management practices, herd/flock size, and experience of farmers contributed significantly to the presence of animal health problems, in particular related to the physical, respiratory and digestive systems. Animal health management was usually conducted in traditional ways. Among different livestock species farms, cattle farms (cattle median BDPI: 45; IQR: 35–55) practised better biosecurity than other livestock species farms (i.e. small ruminant and village chicken farms) (small ruminant and village chicken BDPI: 10; IQR: 0–20). Interestingly, the ownership groups (i.e. rearing singly or multispecies) did not show any impact on biosecurity and disease prevention index of the farms.

**Conclusions:**

This study identified good practice households and these findings will be useful for designing intervention trials to improve the production and health outcomes evaluated in this study.

**Supplementary Information:**

The online version contains supplementary material available at 10.1186/s12917-022-03175-x.

## Background

Livestock production is one of the main income sources for rural households in developing countries and is often central to families’ livelihoods [1–3]. Therefore, understanding the factors influencing livestock production on small scale farms is essential if interventions to increase farmer income are considered [4, 5]. However, animals frequently serve multiple purposes within a household, such as the provision of meat, milk and manure fertiliser, in particular if more than one livestock species is kept on a farm [6–8]. Unfortunately most research studies have concentrated on a single livestock species, ignoring the interactions between a household’s different livestock enterprises, and associations between multi-species rearing and factors such as health management or income generation [9]. For example, livestock research in Myanmar focussed on separate agricultural enterprises without evaluating different livestock rearing activities within individual households or investigating a single disease and did not report the relative significance to other species within the same households [10, 11]. Thus, conducting research that focusses on the linkages, constraints and opportunities within a household’s entire livestock rearing efforts will provide opportunities for more integrated, efficient and relevant strategies for improving livestock production.

In this study we describe livestock husbandry practice, health problems, health management practices and income generated by farmers owning single species or combinations of cattle, small ruminants and/or village chickens in the Central Dry Zone (CDZ) of Myanmar. We then develop a biosecurity and livestock disease prevention index that can be compared between livestock species, estimate the income generated from livestock productions and identify livestock management factors influencing both these parameters. Thus, our study focused on ‘benefits’ (i.e. income) and ‘challenges’ (i.e. management of health and biosecurity) from raising livestock by smallholders in the CDZ.

## Results

### Ownerships

According to our sample size calculation, seven households owning each of the three livestock species in each of the 40 villages, representing 280 households for each species and a total of 840 households was aimed to be collected. However, due to multispecies rearing practice and random sampling for each ownership group, fewer individual households were surveyed, A total 613 household owners were interviewed, with cattle being raised in 382, small ruminants in 303, and village chicken in 327 households. Out of 613 participants, 49.8% (95%CI: 44.2–55.4) were male farmers, and 50.2% (95%CI: 44.6–55.9) were female. From the data, 62.3% of survey households reared cattle, followed by village chicken (53.3% of 613 households) and small ruminants (49.4% of 613 households). The median herd sizes raised in the surveyed households were 4 (IQR: 2–7) cattle, 30 (IQR: 15–41) small ruminants, and 10 (IQR: 5–18) village chicken. Of the 613 households, 19.6% of households had cattle only, 18.9% of households kept cattle and village chicken, 16.8% of households raised small ruminant only, 15.5% of households raised cattle, small ruminant, and village chicken together, 12.2% of households had village chicken only, 9.2% of households had cattle and small ruminants and 7.8% of households raised small ruminant and village chicken.

### Income generated from livestock sales

Total income from livestock sold was estimated for the two-year period before the interview. To understand the profit out of each livestock sale in CDZ, the total income generated from each livestock species (cattle, small ruminant and village chicken) sold was calculated by multiplying the total number of animals sold within the 2 years with median market price over that period for each livestock species. If the farmers sold more than one livestock species, the calculation was done for each livestock species and the total income from livestock sales was derived by the sum of the income from all livestock sales. Median market prices of livestock species animals were obtained from seasonal sale prices specified by farmers over the last 2 years before the interview (considering the sex and if animals were juvenile or adult). There was some fluctuation in market prices of cattle and small ruminants across different seasons, but minimal seasonal variation for village chicken prices (Table 1). Therefore, we used the median value of market price regardless of the seasons and age groups assuming all animals sold were adult with median market price.

**Table 1:** Seasonal variation of sale prices reported by cattle, small ruminant and village chicken farms in the CDZ of Myanmar (Conversion rate US$ 1.0 = 1032.7 MMK) (http://usd.fxexchangerate.com/mmk-2014_12_31-exchange-rates-history.html)

Price	Summer (US$)			Rainy season (US$)			Winter (US$)		
	Offspring	Adult female	Adult male	Offspring	Adult female	Adult male	Offspring	Adult female	Adult male
**Cattle**									
*Minimum*	53.3	67.8	77.5	53.3	125.9	77.5	53.3	53.3	77.5
*Median*	290.5	338.9	503.5	290.5	387.3	677.8	290.5	377.7	542.3
*Maximum*	1355.7	1162.0	1549.3	1452.5	2711.3	3776.5	1355.7	871.5	1500.9
*IQR*	193.7–484.2	242.1–435.8	387.3–677.8	242.1–484.2	266.3–484.2	411.5–871.5	200.9–496.3	242.1–484.2	387.3–774.7
**Small ruminant**									
Minimum	14.5	14.5	38.7	29.1	14.5	19.4	29.1	19.4	33.9
*Median*	45.5	58.1	67.8	38.7	48.4	58.1	38.7	53.3	58.1
*Maximum*	67.8	484.2	968.3	58.1	774.7	968.3	58.1	774.7	968.3
*IQR*	29.1–58.1	48.4–77.5	48.4–96.8	29.1–42.6	43.6–72.6	48.4–96.8	30.3–50.6	38.7–77.5	43.6–96.8
**Village chicken**									
*Minimum*	N/A	1.9	1.9	N/A	1.9	1.9	N/A	1.9	1.9
*Average (Median)*	N/A	4.4	4.4	N/A	4.4	4.4	N/A	4.4	4.4
*Maximum*	N/A	43.6	43.6	N/A	43.6	11.6	N/A	8.7	11.6
*IQR*	N/A	3.9–4.8	3.9–4.8	N/A	3.9–4.8	3.9–4.8	N/A	3.9–4.8	3.9–4.8

To explore demographic and husbandry factors influencing income derived from livestock sales by comparing within the livestock enterprise, we calculated the median income for each livestock species and categorised income into three groups: no income (US$ 0 for all livestock ownership), less or equal to the median income, i.e. low (<US$ 450 for cattle ownership; <US$ 533 for small ruminant ownership; <US$ 373 for village chicken ownership), and larger than the median income, i.e. high (>US$ 450 for cattle ownership; >US$ 533 for small ruminant ownership; >US$ 373 for village chicken ownership).

### Livestock health problems

**Fig. 1:**
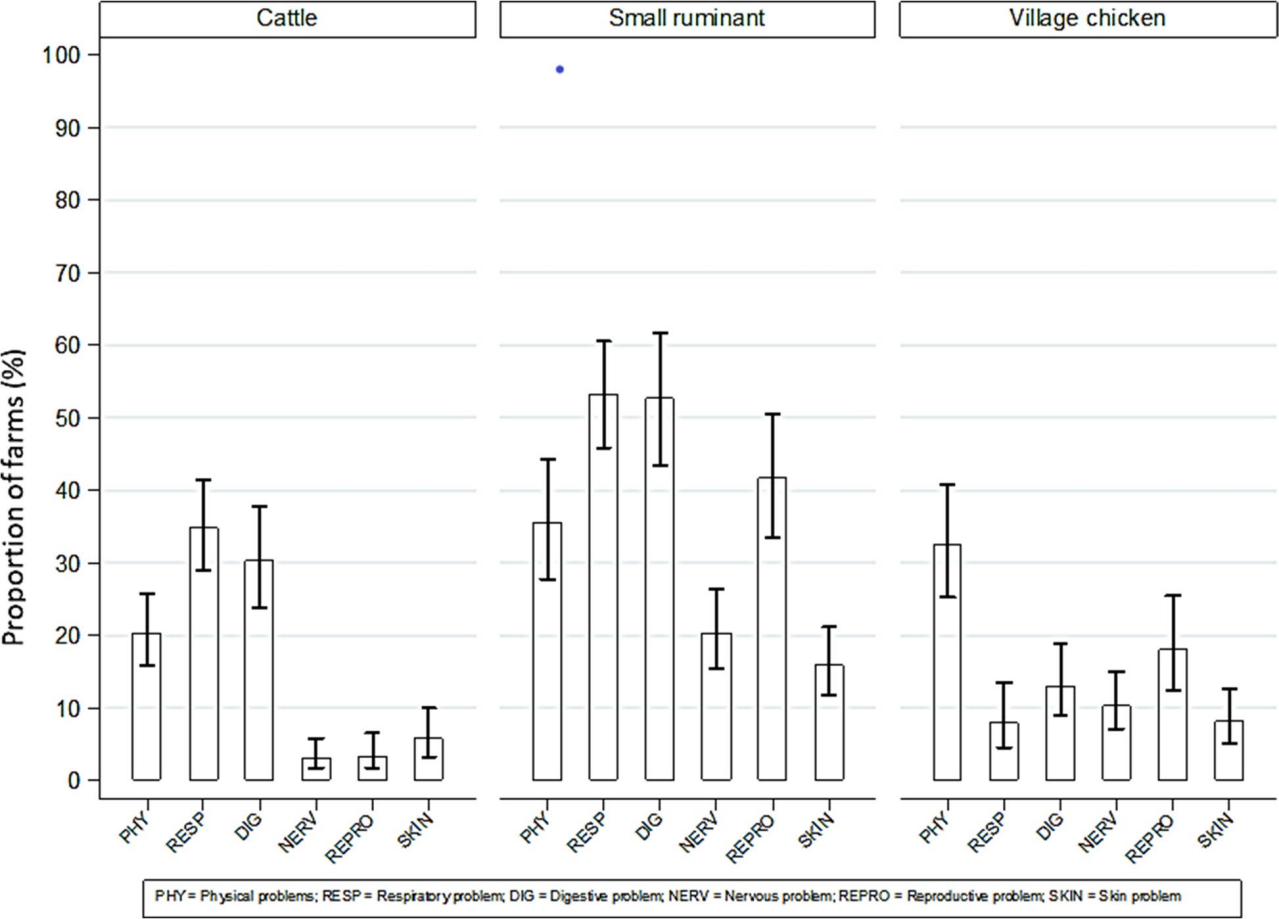
Proportion of cattle, small ruminant and village chicken farms reporting the presence of syndromic health problems within the last year before the interviews in the CDZ of Myanmar

Physical problems (lameness, retarded growth, weakness, frequent recumbency in ruminants, twisted head and neck in village chickens) were reported in 23.3% of cattle, 35.6% of small ruminant and 32.5% of village chicken households. Respiratory disorders (coughing, sneezing, nasal discharge or other breathing problems) were reported in 40.0% of cattle, 53.3% of small ruminant and 7.9% of village chicken households, and digestive problems (drooling or sores in the mouth, unwillingness to eat or anorexia, constipation or straining to defecate, abdominal pain, diarrhoea) were common across all livestock species and were reported in 34.8% of cattle, 52.6% of small ruminant and 13.0% of village chicken households. Overall, small ruminant farmers reported the highest frequency of livestock health problems across all body system-related categories compared to cattle and village chicken farmers. In particular, reproductive problems were more commonly observed in small ruminants compared to the other livestock species (Fig. 1).

Respiratory and digestive disorders in cattle were more common in adults than in offspring (*p* < 0.05). Apart from digestive problems, which occurred most frequently in small ruminant offspring, all other health problems occurred more frequently in adult small ruminants (*p* < 0.05). Problems of the digestive and the nervous system were more common in chicks than in older birds (*p* < 0.05) (Table 2).

**Table 2:** Proportion of households reporting different animal health problems on cattle, small ruminant and village chicken farms in the CDZ of Myanmar

Body system affected	Age group	Cattle		Small ruminants		Village chickens	
		N	% (95% CI)	N	% (95%CI)	N	%(95%CI)
Physical disorders	Offspring	158	7.0 (3.9–12.2)	275	18.8 (12.1–27.9)*	218	25.5 (18.9–33.3)
	Adult female	243	15.2 (11.2–20.4)	291	33.1 (24.5–43.0)*	313	28.4 (21.1–36.9)
	Adult male	234	16.1 (11.3–22.3)	268	21.5 (15.6–28.7)*	209	26.3 (18.8–35.4)
Respiratory disorders	Offspring	158	12.0 (7.8–18.2)*	275	23.4 (19.0–28.4)*	218	5.6 (2.7–11.3)
	Adult female	243	26.3 (21.2–32.3)*	291	48.9 (41.5–56.4)*	275	3.4 (1.6–7.0)
	Adult male	234	30.2 (23.0–38.6)*	268	36.5 (29.1–44.5)*	185	6.4 (2.8–14.2)
Digestive disorders	Offspring	158	5.1 (2.5–9.9)*	275	45.7 (36.5–55.2)*	218	13.1 (8.6–19.6)*
	Adult female	243	23.9 (18.9–29.7)*	291	38.3 (32.0–44.9)*	275	9.4 (5.7–15.2)*
	Adult male	234	32.5 (24.4–41.8)*	268	25.9 (19.9–33.0)*	185	7.2 (4.0–12.5)*
Nervous disorders	Offspring	158	0.6 (0.01–4.4)	275	6.3 (3.4–11.2)*	218	10.2 (6.9–14.7)*
	Adult female	243	3.7 (1.9–7.0)	291	13.6 (9.6–19.0)*	275	2.2 (0.8–6.0)*
	Adult male	234	1.9 (0.6–5.9)	268	8.3 (5.5–12.3)*	185	3.0 (1.1–8.2)*
Skin	Offspring	158	3.8 (1.7–8.3)	275	7.7 (5.0–11.6)*	218	5.5 (2.5–11.5)
	Adult female	243	2.9 (1.4–5.9)	291	13.3 (9.5–18.4)*	275	4.3 (2.1–8.6)
	Adult male	234	5.3 (2.9–9.5)	268	9.5 (5.9–14.9)*	185	6.2 (2.9–12.5)
Reproductive disorders	Offspring	158	N/A	275	N/A	218	N/A
	Adult female	243	5.4 (3.1–9.0)	291	41.6 (33.3–50.4)*	275	16.2 (10.8–23.5)*
	Adult male	234	0.0	268	0.3 (0.1–2.6)*	185	3.8 (1.6–8.7)*

(* = *p*< 0.05, significant difference between age groups within species)

Grazing practices, herd sizes, biosecurity and livestock disease prevention index were associated with health problems in different body systems (Table 3). The occurrence of respiratory and digestive disorders in cattle was associated with larger herd sizes (i.e. the comparison of raising 1–3 cattle vs 4–6 cattle vs more than 6 cattle) (*p* < 0.001), while physical disorders were more commonly observed on cattle farms that practise grazing (*p* = 0.022). The only health issue associated with different livestock species rearing combinations was digestive problems in village chickens, which occurred less frequently in birds in households that kept village chickens together with other livestock species, compared to households only keeping village chickens (*p* = 0.025). Surprisingly, more experienced small ruminant farmers practised poorer biosecurity and disease prevention than less experienced farmers. Also, observing digestive problems in small ruminants resulted in implementing better biosecurity and livestock disease prevention practices (*p* < 0.05) (Tables 3 and 4).

**Table 3:** Univariable analysis of factors associated with the reported occurrence of different livestock health problems on cattle, small ruminant and village chicken farms in the CDZ of Myanmar (each health problem-species combination represents a separate analysis)

Variables	Categories	N	Percentage (%)		OR	***p***-value	Wald test
			No	Yes			
**Outcome variable: Physical disorders in cattle**							
Yes – 74 (20.3%); No – 308 (79.7%)							
Herd size	Low (1–3)	382	44.2	30.8	1		0.0294
	Medium (4–6)		33.6	29.9	1.3 (0.6–3.0)	0.568	
	High (> 6)		22.2	39.3	2.5 (1.1–5.7)	0.024	
Practise grazing	No	382	26.4	13.2	1		**–**
	Yes		73.6	86.8	2.4 (1.1–4.9)	0.022	
**Outcome variable: Respiratory disorders in cattle**							
Yes – 118 (34.9%); No – 264 (65.1%)							
Herd size	Low (1–3)	382	53.1	19.8	1		< 0.0001
	Medium (4–6)		25.4	46.8	4.9 (2.8–8.7)	< 0.0001	
	High (> 6)		21.5	33.4	4.2 (2.4–7.2)	< 0.0001	
**Outcome variable: Digestive disorders in cattle**							
Yes – 109 (30.4%); No – 273 (69.6%)							
Herd size	Low (1–3)	382	48.3	25.7	1		0.0083
	Medium (4–6)		31.1	36.8	2.2 (1.0–4.8)	0.042	
	High (> 6)		20.5	37.5	3.4 (1.6–7.3)	0.002	
**Outcome variable: Digestive disorders in small ruminant**							
Yes – 146 (52.6%); No – 157 (47.4%)							
BDPI	Poor	303	34.7	19.9	1		0.0308
	Low		35.5	38.0	1.9 (1.1–3.2)	0.024	
	High		29.8	42.2	2.5 (1.2–5.0)	0.013	
**Outcome variable: Digestive disorders in chicken**							
Yes – 45 (13.0%); No – 282 (87.0%)							
Type of animal rearing in the same household	Chicken only	327	18.1	42.1	1		0.0250
	Cattle + Chicken		33.3	31.1	0.4 (0.2–1.0)	0.053	
	Small ruminant + Chicken		21.4	10.4	0.2 (0.1–0.6)	0.007	
	Cattle + Small ruminant + Chicken		27.2	16.4	0.3 (0.1–0.7)	0.008	
**Outcome variable: Physical disorders in chicken**							
Yes – 98 (32.5%); No – 229 (67.5%)							
BDPI	Poor	327	45.6	22.6	1		0.0047
	Low		32.0	34.6	2.2 (1.0–4.7)	0.046	
	High		22.4	42.7	3.8 (1.8–8.2)	0.001

**Table 4:** Factors associated with the reported occurrence of different livestock health problems on cattle, small ruminant and village chicken farms in the CDZ of Myanmar (each health problem-species combination represents a separate analysis)

Variables	Categories	N	Percentage (%)		Odds ratio	***p***-value	Wald test
			No	Yes			
**Outcome variable: Physical disorders in cattle**							
Yes – 74 (20.3%)							
No – 308 (79.7%)							
Practise grazing	No	382	26.4	13.2	1		–
	Yes		73.6	86.8	2.4 (1.1–4.9)	0.022	
**Outcome variable: Respiratory disorders in cattle**							
Yes – 118 (34.9%)							
No – 264 (65.1%)							
Herd size	Low (1–3)	382	53.1	19.8	1		< 0.0001
	Medium (4–6)		25.4	46.8	4.9 (2.8–8.7)	< 0.0001	
	High (> 6)		21.5	33.4	4.2 (2.4–7.2)	< 0.0001	
**Outcome variable: Digestive disorders in cattle**							
Yes – 109 (30.4%)							
No – 273 (69.6%)							
Herd size	Low (1–3)	382	48.3	25.7	1		0.0083
	Medium (4–6)		31.1	36.8	2.2 (1.0–4.8)	0.042	
	High (> 6)		20.5	37.5	3.4 (1.6–7.3)	0.002	
**Outcome variable: Digestive disorders in small ruminant**							
Yes – 146 (52.6%)							
No – 157 (47.4%)							
BDPI	Poor	303	34.7	19.9	1		0.0308
	Low		35.5	38.0	1.9 (1.1–3.2)	0.024	
	High		29.8	42.2	2.5 (1.2–5.0)	0.013	
**Outcome variable: Physical disorders in chicken**							
Yes – 98 (32.5%)							
No – 229 (67.5%)							
BDPI	Poor	327	45.6	22.6	1		0.0047
	Low		32.0	34.6	2.2 (1.0–4.7)	0.046	
	High		22.4	42.7	3.8 (1.8–8.2)	0.001	
**Outcome variable: Digestive disorders in chicken**							
Yes – 45 (13.0%)							
No – 282 (87.0%)							
Type of animal rearing in the same household	Chicken only	327	18.1	42.1	1		0.0250
	Cattle + Chicken		33.3	31.1	0.4 (0.2–1.0)	0.053	
	Small ruminant + Chicken		21.4	10.4	0.2 (0.1–0.6)	0.007	
	Cattle + Small ruminant + Chicken		27.2	16.4	0.3 (0.1–0.7)	0.008	

### Biosecurity and livestock disease prevention

**Fig. 2:**
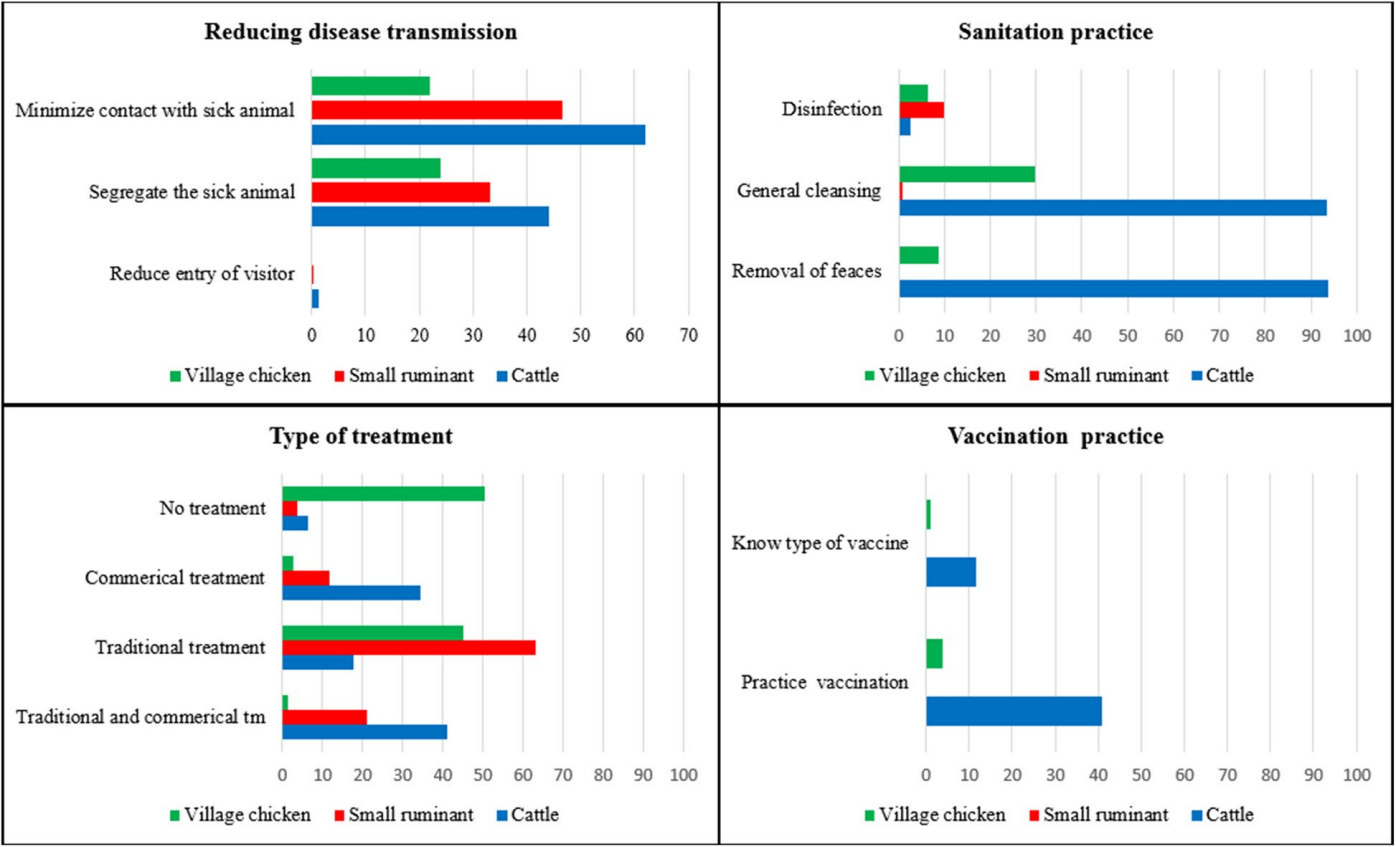
Proportion of cattle, small ruminant and village chicken farms in the CDZ of Myanmar conducting livestock treatment and vaccinations of livestock and implementing disease prevention and sanitation measures

More than half of village chicken owners did not treat sick chickens, while only 6.6 and 3.9% of cattle and small ruminant owners did not treat their sick animals. If treatment was conducted, the majority of the small ruminant (> 60%) and village chicken owners (~ 50%) relied on traditional medicine, while the majority of cattle farmers (> 60%) used veterinary health care providers alone or in combination with traditional medicine (Table 5) (Fig. 2). Approximately 69.7% of village chicken and 63.3% of small ruminant owners did not implement any specific biosecurity measures to reduce the spread of livestock diseases, in contrast to 28.7% of cattle owners. The most common disease control approach was the segregation of sick animals (43.9, 34.0 and 24.6% of cattle, small ruminant, and village chicken owners respectively), usually until recovery.

**Table 5:** Health management and biosecurity practices conducted by cattle, small ruminant and village chicken farms in the CDZ of Myanmar

No.	Management practices	Categories	Cattle		Small ruminants		Village chickens		Comparison between different species	
			N	% (95% CI)	N	% (95% CI)	N	% (95% CI)	F-statistics	*p*
1.	Treatment of sick animals	Not conducted	382	6.6 (4.3-10.0)	303	3.9 (2.1-7.2)	327	53.4 (46.1-60.6)	72.5	<0.0001
		Traditional treatment^a^		17.7 (13.8-22.7)		63.4 (53.1-72.5)		43.1 (36.3-50.1)		
		Veterinary treatment		34.6 (27.8-42.0)		11.7 (8.2-16.5)		2.0 (0.9-4.6)		
		Both		41.1 (33.2-49.4)		21.0 (14.1-30.1)		1.5 (0.6-4.0)		
2.	Implementation of biosecurity measures on the farm	Yes	382	71.3 (64.7-77.1)	303	36.7 (29.6-44.3)	327	30.3 (24.1-37.4)	58.3	<0.0001
		No		28.7 (23.0-35.3)		63.3 (55.7-70.4)		69.7 (62.6-75.9)		
3.	Restrict entry of visitors to farms	Yes	382	1.2 (0.4-3.5)	303	0.4 (0.1-2.7)	327	0	199.3	<0.0001
		No		98.8 (96.5-99.6)		99.6 (97.3-99.9)		100		
4.	Disinfection conducted on the farm	Yes	382	2.7 (1.6-4.3)	303	9.0 (5.6-14.1)	327	3.6 (1.8-7.2)	7.5	0.0017
		No		97.3 (95.7-98.4)		91.0 (85.9-94.4)		96.4 (92.8-98.2)		
5.	Segregation of sick animals on the farms	Yes	382	43.9 (38.1-49.9)	303	34.0 (25.9-43.1)	327	24.6 (18.0-32.6)	6.3	0.0003
		No		48.6 (42.0-55.2)		60.6 (52.7-67.9)		66.4 (58.1-73.8)		
		Don't know		7.5 (4.9-11.4)		5.4 (3.4-8.6)		9.0 (6.4-12.5)		
6.	Segregation of sick animals until recovery	Yes	382	44.1 (38.3-50.0)	303	33.2 (25.1-42.4)	327	24.0 (17.8-31.4)	6.4	0.0004
		No		48.4 (41.9-55.0)		59.8 (52.0-67.2)		67.8 (59.6-75.0)		
		Don't know		7.5 (4.9-11.4)		7.0 (4.6-10.6)		8.2 (5.6-11.9)		

^a^E.g.local herbal drugs, indigenous way of treating

**Fig. 3:**
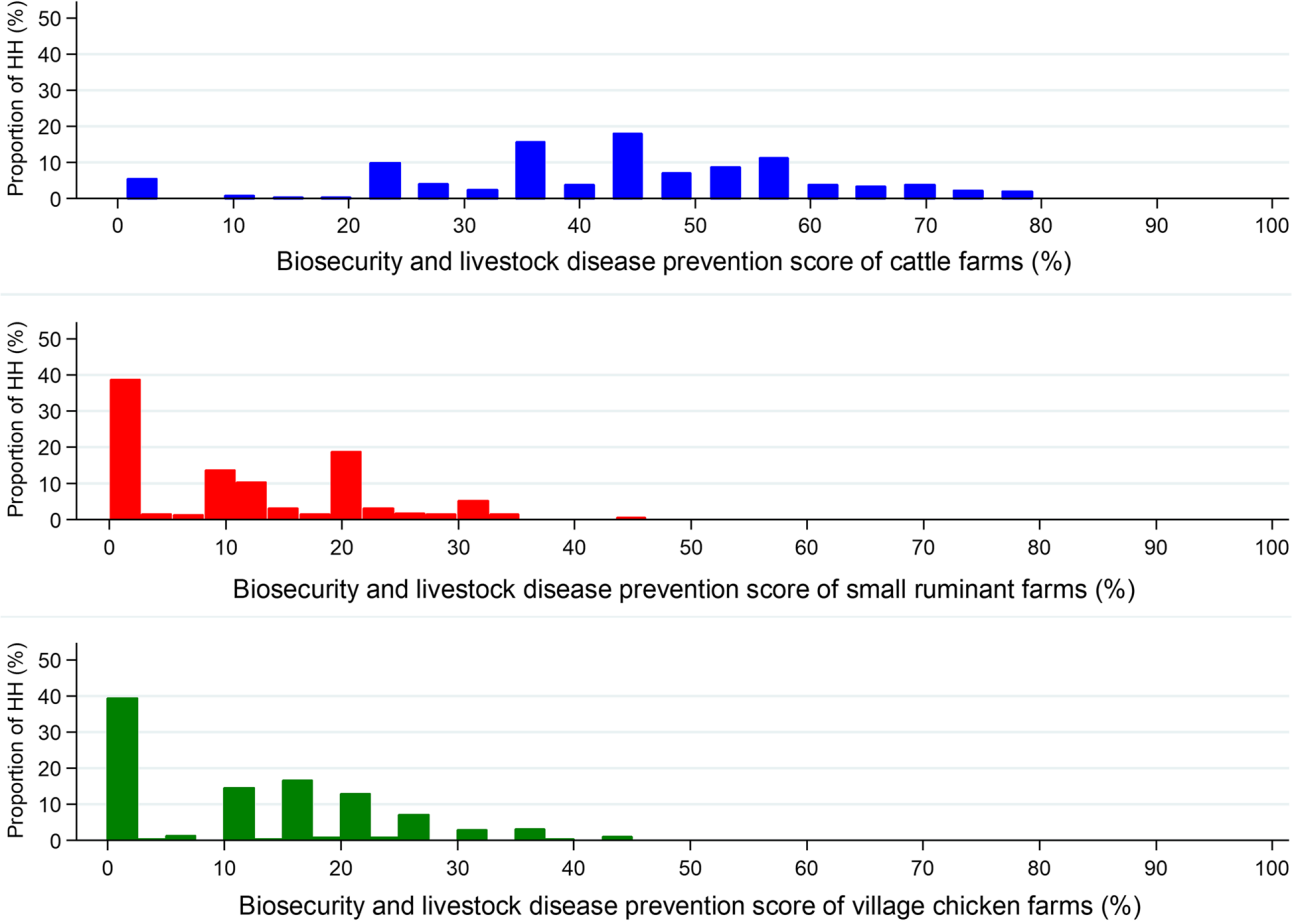
Proportion of cattle, small ruminant and village chicken farms with different biosecurity and livestock disease prevention indices in the CDZ of Myanmar

Cattle owners conducted better biosecurity and disease prevention practices (cattle median BDPI: 45; IQR: 35–55) compared to small-ruminant and village chicken farmers (small ruminant and village chicken BDPI: 10; IQR: 0–20) (Fig. 3).

**Fig. 4:**
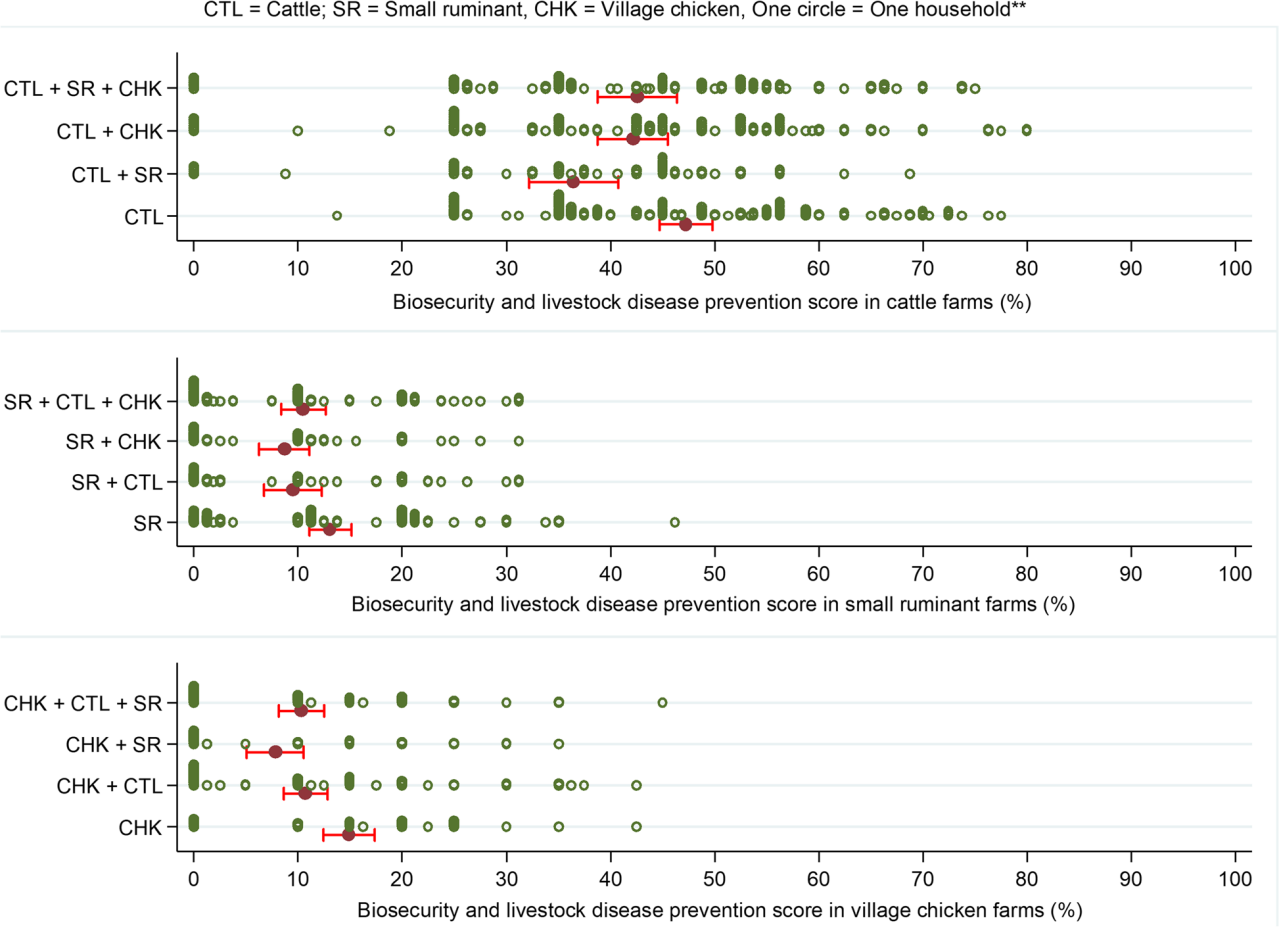
Distribution of biosecurity and livestock disease prevention indices on farms raising combinations of cattle, small ruminant and village chicken in the CDZ of Myanmar

The biosecurity and livestock disease prevention index (BDPI) was similar within each of the three livestock ownership groups, when cattle, small ruminants or village chickens were kept in combination with other livestock species (Fig. 4).

Better biosecurity and livestock disease prevention practices were implemented by cattle and village chicken farmers with more than 5 years of experience in raising these livestock species, with farms with a longer history of keeping animals having 1.9 (village chickens) and 3.0 (cattle) times the odds of having a greater BDPI score than those with a shorter history of ownership (Table 6).

**Table 6:** Factors affecting biosecurity and disease prevention indexes (BDPI) on cattle, small ruminant and village chicken farms in the CDZ of Myanmar

Variables	Categories	N	% of households in BDPI category			Odds ratio	***p***-value	Wald test
			Poor	Low	High			
**Outcome variable: BDPI in cattle farm**								
Poor (0%) – 20 (5.0%)								
Low (1–45%) – 197 (51.1%)								
High (> 45%) – 165 (43.9%)								
Duration of rearing cattle	< 5 years	382	22.1	11.9	4.7	1		–
	> 5 years		77.9	88.1	95.3	3.0 (1.0–9.0)	0.049	
**Outcome variable: BDPI in small ruminant farm**								
Poor (0%) – 79 (26.9%)								
Low (1–12.5%) – 117 (36.8%)								
High (> 12.5%) – 107 (36.3%)								
Duration of rearing sheep	< 5 years	303	77.9	86.5	94.7	1		–
	> 5 years		22.1	13.5	5.3	0.3 (0.2–0.6)	< 0.0001	
**Outcome variable: BDPI in village chicken farm**								
Poor (0%) – 126 (38.1%)								
Low (1–15%) – 106 (32.9%)								
High (> 15%) – 95 (29.0%)								
Type of animal reared	Village chicken only	327	14.8	18.5	32.7	1		0.0020
	Cattle + Village chicken		25.4	42.2	32.7	0.6 (0.2–1.7)	0.336	
	Small ruminant + Village chicken		34.1	11.6	10.8	0.2 (0.1–0.5)	0.001	
	All 3 spp.		25.7	27.8	23.7	0.5 (0.2–1.3)	0.134	
Duration of rearing village chicken	< 5 years	327	31.2	22.4	16.0	1		–
	> 5 years		68.8	77.6	84.0	1.9 (1.3–3.0)	0.002	

### Income generated from livestock sales

Of the 613 farmers surveyed, 435 farmers (69.1%) reported that they sold animals in the 2 years before the interview, while 178 farmers (30.9%) did not sell animals. Amongst the latter, households that did not sell animals represented 36.9% of cattle, 18.9% of small ruminant and 23.2% of village chicken owners.

**Fig. 5:**
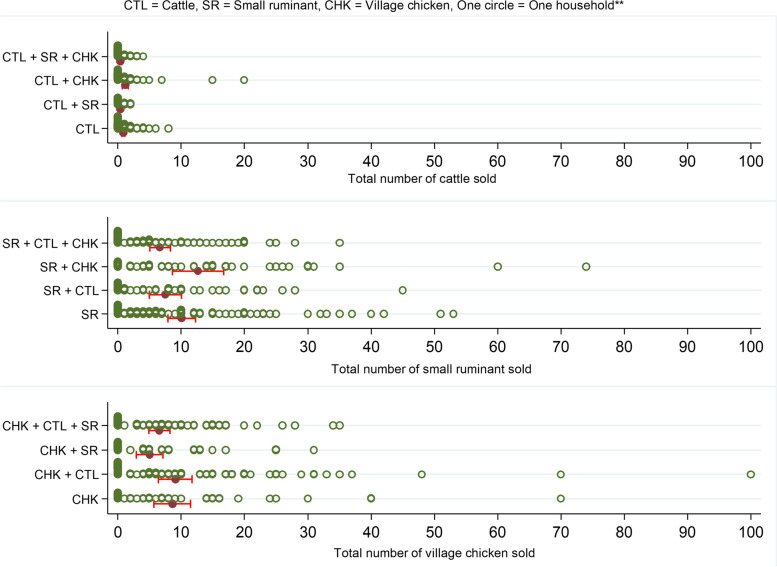
Number of animals sold in two years before the interview on farms raising combinations of cattle, small ruminant and village chicken in the CDZ of Myanmar (red horizontal bar indicates the mean of the number of animal sales with 95% confidence interval)

Excluding the households with no history of sale, the patterns of sales were similar for cattle and village chicken owning households that sold livestock across different livestock ownership groups, with a median of 1–2 cattle and 8–9 village chicken being sold in the past 2 years before the interviews (Fig. 5). However, the median number of small ruminants sold varied across different livestock ownership groups with sales: 10 sheep or goats on small ruminant only farms, 8 on farms with cattle and small ruminants, 14 on farms with small ruminants and village chickens, and 7 on farms with cattle, small ruminant and village chicken.

**Fig. 6:**
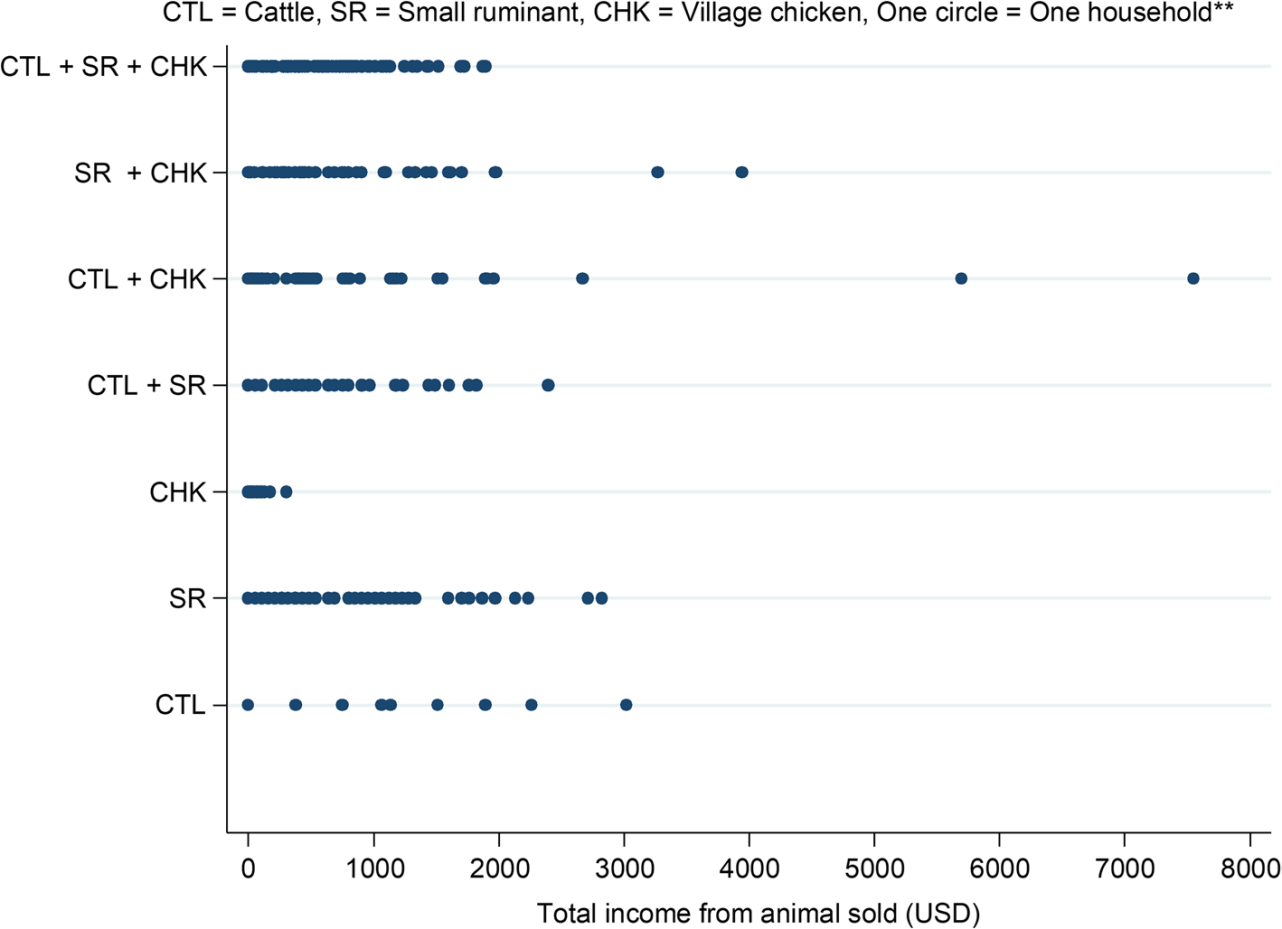
Total income generated from livestock sales within the last two years before the interviews on farms raising different combinations of livestock species in the CDZ of Myanmar

The distribution of income from farms with livestock sales is shown in Fig. 6. Households with only village chickens generated the lowest income. The median income (IQR) generated in village chicken, small ruminant and cattle only farms over the two-year period from sales of livestock was 34.9 USD (21.8–69.7), 532.6 USD (266.3–905.4) and 755.3 USD (377.7–910.2) respectively. Households keeping village chickens or small ruminants with other livestock species were more likely to earn higher income from livestock sales, whereas cattle households raising small ruminants and/or chickens reported lower income from livestock sales (Table 7).

**Table 7:** Total income generated from livestock sale within the past 2 years before the interview on farms raising combinations of cattle, small ruminant and village chicken in the CDZ of Myanmar (http://usd.fxexchangerate.com/mmk-2014_12_31-exchange-rates-history.html)

Average income from livestock sale (US$)	Cattle	Small ruminant	Village chicken	Cattle + Small ruminant	Cattle + Village chicken	Small ruminant + Village chicken	Cattle + Small ruminant + Village chicken
*Minimum*	377.7	53.3	4.4	53.3	8.7	8.7	13.1
*Median*	755.3	532.6	34.9	585.8	386.4	639.1	556.6
*Maximum*	3021.2	2822.7	69.7	2396.6	7553.0	3941.1	1894.6
*IQR*	377.7–910.2	266.3–905.4	21.8–69.7	334.1–1171.7	56.7–755.3	266.3–1093.7	279.4–907.6

Higher income from livestock sales occurred for cattle and village chicken farmers when additional livestock species were kept within the same household. In small ruminant-owning households, greater livestock income occurred in herds/flocks that experienced respiratory or digestive problems (Table 8).

**Table 8:** Factors associated with the income from livestock sales on cattle, small ruminant and village chicken farms in the CDZ of Myanmar *This cut-off represent the median income from the sale of animals of this livestock species

Variables	Categories	N	% of households in income category			Odds ratio	***p***-value	Wald test
			Low	Medium	High			
**Outcome variable: Income generated from selling cattle**								
No income (US$ 0) – 128 (36.9%)								
Low (< US$ 450) – 127 (32.2%)*								
High (> US$ 450) – 127 (30.9%)								
Type of animal reared	Cattle only	382	56.0	19.5	22.6	1		0.0003
	Cattle + Small ruminant		13.2	9.7	22.5	4.1 (1.4–12.0)	0.013	
	Cattle + Village chicken		20.6	40.8	25.9	3.1 (1.7–5.9)	0.001	
	Cattle + Small ruminant + Village chicken		10.2	30.0	29.1	5.1 (2.5–10.3)	< 0.0001	
Reproductive disorders	No	382	99.3	96.3	93.9	1		–
	Yes		0.7	3.7	6.1	4.5 (2.2–9.3)	< 0.0001	
**Outcome variable: Income generated from selling small ruminant**								
No income (US$ 0) – 55 (18.9%)								
Low (< US$ 533) – 131 (39.9%)*								
High (> US$ 533) – 117 (41.1%)								
Digestive disorders	No	303	63.4	49.7	37.8	1		–
	Yes		36.6	50.3	62.2	0.6 (0.1–1.0)	0.023	
Reproductive disorders	No	303	75.9	63.8	45.0	1		–
	Yes		24.1	36.2	55.0	0.8 (0.3–1.3)	0.001	
**Outcome variable: Income generated from selling village chicken**								
No income (US$ 0) – 72 (23.2%)								
Low (< US$ 373) – 129 (39.7%)*								
High (> US$ 373) – 126 (37.1%)								
Types of animal reared	Village chicken only	327	32.4	34.5	0	1		< 0.0001
	Cattle + Village chicken		37.8	27.6	35.9	3.2 (1.8–5.5)	< 0.0001	
	Small ruminant + Village chicken		11.1	14.5	31.2	7.5 (3.6–15.3)	< 0.0001	
	All 3 spp.		18.7	23.4	32.8	4.8 (2.3–10.3)	< 0.0001	

### Main income sources

**Fig. 7:**
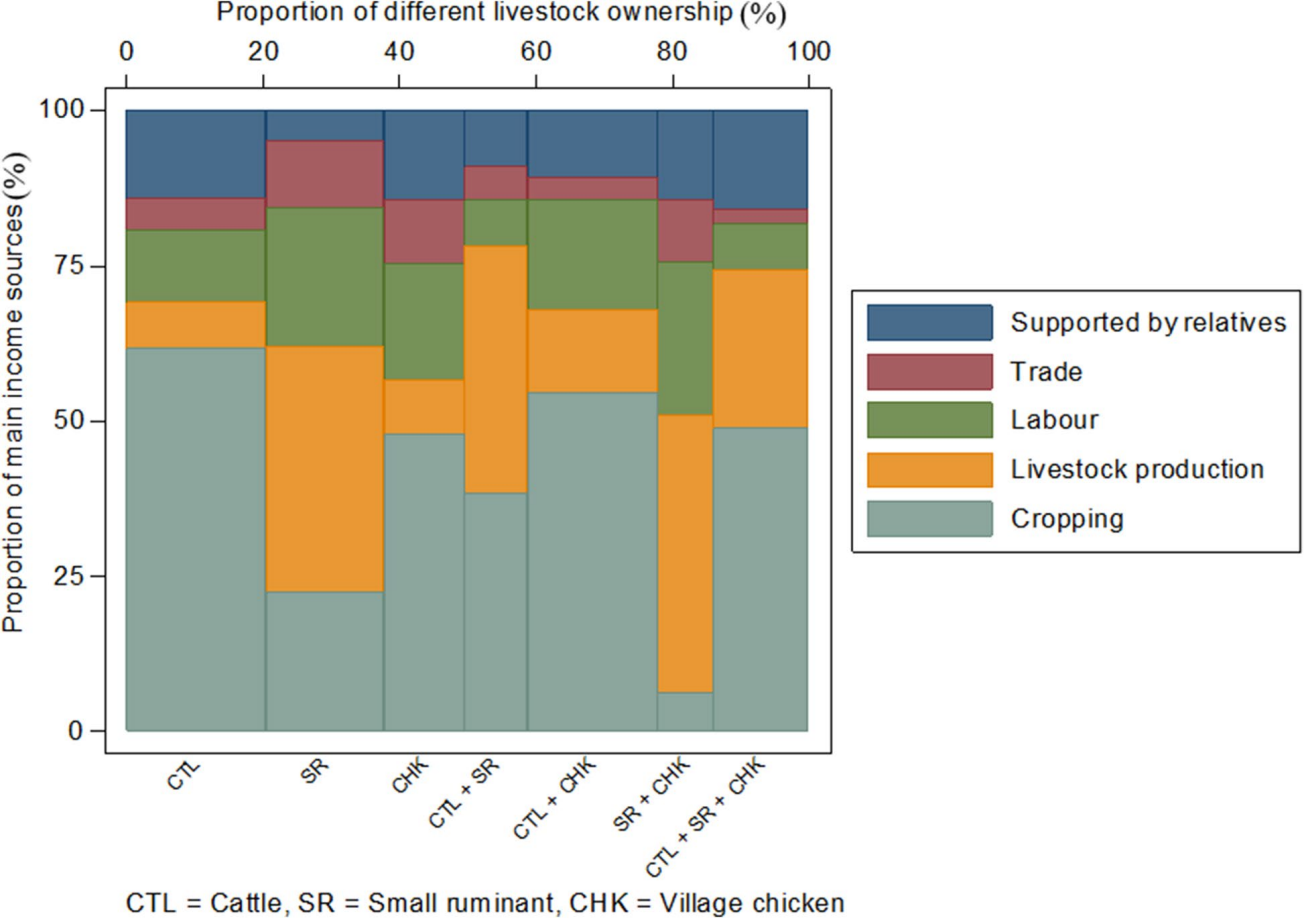
Proportion of main income sources for farms raising different combinations of livestock species in the CDZ of Myanmar (width of columns indicates the proportion of farms owning each combination of livestock species)

A total of 590 respondents provided information on all their household income sources: 43.2% of farmers obtained their highest income from cropping; 23.1% from livestock production; 15.6% from employment; 11.7% from support by relatives (‘remittances’) and 6.4% from trade (Fig. 7). The top income sources for different livestock ownership are shown in Fig. 7. For all cattle owning households (keeping cattle only or in combination with other livestock species) cropping was the main income source. For all small ruminant farmers (keeping small ruminants only or in combination with other species), livestock production (and sales) was the dominant income source. When village chickens were raised alone or with cattle, cropping was the main income source, but when village chickens were kept with small ruminants, livestock sales were the top income source.

## Discussion

In this research we identified key constraints to livestock production and health, and thereby farmer livelihoods from small-scale cattle, goat/sheep and village chicken production in the CDZ of Myanmar. We adopted a syndromic approach to summarize health problems in order to avoid the use of intensive resources and multiple panels of diagnostic tests and to reduce potential information bias associated with a survey team’s clinical expertise in diagnosing livestock diseases. This approach has been used before in Myanmar for village chicken health problems [12], but not to date on small ruminant or cattle farms.

‘Physical’ health problems were most commonly observed in village chickens. This syndromic category included signs such as twisted head and neck, which are consistent with Newcastle disease, a common and important disease of poultry in the CDZ [13–15]. A similar phenomenon was observed in small ruminant-owning households that reported digestive problems in their animals. This suggests that farmers do respond to disease events, even those owning species that principally rely on ‘traditional’ remedies and have poorer access to formal health services. This awareness suggests that additional government support for disease prevention would likely be welcomed by farmers and have a beneficial effect on further disease control.

In cattle and small ruminants, ‘respiratory’ and ‘digestive’ signs were most common, followed by ‘reproductive’ signs in small ruminants. Similar observations were made by [16] (Submitted). The reported prevalence of health problems in cattle was lower than in the two other livestock species under study. This might be explained by the fact that cattle are normally the livestock species with the highest market value and for that reason cattle farmers might be more willing to spend money in the treatment of, or aimed at improving biosecurity and disease prevention for these species.

Our study showed the pattern of clinical syndromes varied between different-sized cattle holdings, with digestive and respiratory problems reported more frequently in larger herds compared to smaller ones (i.e. the comparison of raising 1–3 cattle vs 4–6 cattle vs more than 6 cattle). It is possible that increasing herd/flock size challenges farmers’ management skills, limiting the success or sustainability of keeping greater livestock numbers [17–19]. Additionally, increased trading as a household’s livestock holdings grow may present new disease threats (i.e. infectious diseases such as Foot and Mouth Disease, Newcastle disease, Highly Pathogenic Avian Influenza, other bacterial diseases, etc.). Feeding practices were also associated with cattle health in that poor nutrition as a result of animals mainly fed via grazing might increase their disease susceptibility, or this feeding strategy may increase contact with animals outside the household and facilitate disease spread. This information identifies classes of livestock that may warrant more attention from farmers and health services. It suggests extension and support for livestock health and production may benefit from being tailored to different enterprise sizes [7, 20–22], and not assume that a ‘one size fits all’ approach to livestock health for each species is appropriate.

Our previous study highlighted that livestock in CDZ of Myanmar is raised in traditional ways, such as by provision of grazing [23]. The present study extends these findings to the widespread use of traditional medicines to treat health problems. Furthermore, the decision to use ‘commercial veterinary products’ for treating animal diseases is likely to be driven by the value of the animals, explaining why in our study cattle were more often treated with commercial products compared to other species [18, 24]. Our findings of a greater reliance on farmer-sourced, traditional remedies strongly supports anecdotal observations that there is poorer communication between health providers, including government, and goat/sheep and village chicken owners than those keeping cattle. This likely has flow-on effects into poor awareness of cross-species disease transmission risks and biosecurity practices; among different livestock ownership groups, our study noted that biosecurity and disease prevention practices were more common on cattle farms than small ruminant or village chicken farms. Further studies to investigate the factors affecting farmers’ decisions in relation to animal health care are required to inform strategies to improve animal health care provision in the CDZ of Myanmar.

Despite biosecurity and infection control being relevant to the management of all the livestock species covered in our study [25–27], there was considerable variation between livestock enterprises in how well these were practised. In turn, this likely impacts the profitability and sustainability of these different enterprises. Health problems and biosecurity practices were not associated with different livestock ownership combinations on small ruminant and cattle farms. However, on village chicken farms, poor biosecurity practices were more common amongst multispecies-rearing households, as the BDPI was lower when chickens were kept with other livestock species. This suggests farmers preoccupied with other activities were less likely to give attention to village chicken health management, as the chicken is a low capital source of income [28]. This is important in terms of lost opportunities for those households and also identifies a group of households more at risk of potentially important diseases, such as avian influenza. Despite these findings, fewer digestive disorders were actually reported in village chickens in multispecies-rearing households compared to households raising only village chickens. However, it has to be considered that signs of clinical disease in village chickens might have been underreported as they are of lower importance compared to other livestock species in multispecies households.

One of the unexpected findings from our study is that farms with health disorders in cattle and small ruminants were more likely to earn greater income. One explanation could be that the farmers tend to sell unwell animals rather than treat them. This may be a result of poor farmer understanding of disease management or they might not be aware of the benefit of the good health care practice on farms. This especially occurred in small ruminant herds. We recommend further research to describe the associations between an animal’s health status and sale price, and farmer attitudes and knowledge of livestock trading, animal health status and risk of disease spread.

It was interesting to note that about one fifth of small ruminant and one quarter of village chicken households sold no animals in the 2 years preceding our study, despite these species typically being kept to generate cash income. A better understanding is required of the factors that influence livestock sales and hence household income, as increased farmer awareness of market volatility and the most suitable time or age of animals to sell, or improved trading resources and connections with value chains may improve household income.

There were a number of obstacles and potential limitations in our study typical of research in this area. We adopted a syndromic approach to describe occurrence of health problems to overcome the frequent lack of accurate disease diagnosis in the CDZ. To compare management of different livestock species, we developed a summary measure of biosecurity and livestock disease prevention index (BDPI). Even though we tested adjustment and validation of the scores to get reliable data, the index would nonetheless benefit from further validation and evaluation in different management scenarios. Lastly, few farmers kept animal health, production or trading records. Therefore, it was necessary to calculate average market values from the data collected from farmers because it was very hard to get reliable data from individual farmers. Because livestock prices are relatively volatile [10], future longitudinal studies are required to better collect more reliable livestock price and household income data.

## Conclusion

Our study has shown that different livestock enterprises, and combinations thereof, vary in their role in household livelihoods and in terms of constraints they face in the Central Dry Zone of Myanmar. Despite the significance of these enterprises to household incomes, health problems are common. Nonetheless, all livestock systems contained examples of good biosecurity and disease management practices. Households using these methods would serve as leaders in extension programs to improve production and health management. This is likely to be especially important for systems containing comparable species combinations and of similar size, as adopting a ‘one size fits all’ approach to improving production and health would be less likely to address the important nuances in livestock production our study has described. This study identified good practice households and these findings will be useful for designing intervention trials to improve the production and health outcomes evaluated in this study.

## Methods

### Study design

A cross-sectional study using a questionnaire survey was conducted among small-scale farming households (i.e. raising small number of livestock for additional household income with limited farming facilities) owning different livestock species in two administrative areas (townships), Myingyan and Meikhtila, in the CDZ of Myanmar. These two CDZ townships were identified as representative of typical livestock production systems in the CDZ by a research-for-development project investigating livestock production [29]. To identify the representativeness, collecting expert opinion was conducted to observe different criteria (i.e. husbandry practice, poverty level, livelihood of farmers, climate, number of livestock) among the townships in CDZ.

In brief, a two-stage sampling approach was used with villages and households as the primary and secondary sampling units (PSU and SSU) respectively. Data were collected from a total of 40 villages within the two townships. Random sampling with replacement was used to select seven households each owning cattle, small ruminants and village chickens per village, providing a total of 21 households per village, to obtain at least seven households each owning cattle, small ruminant and village chickens. Sample size calculations and random sampling were performed using the Survey Toolbox modules Sample size for 2-stage prevalence survey (http://epitools.ausvet.com.au/content.php?page= SurveyToolbox) [30].

### Questionnaire and data collection

A questionnaire was developed in English and was then translated into the local language (Myanmar). Then for the data entry and analysis, all the data were translated into English. The questionnaire collected information about the livestock kept on each farm, current livestock husbandry practices (14 questions for species), income generated from various sources (18 questions per species), animal health problems (12 questions per species), the management of animal health issues and biosecurity (14 questions per species) in the past 12 months and information on animal sales in the last 2 years. The collected data using this questionnaire was also published in another study [31]. The survey was pilot-tested in two villages in Meikhtila township and the final version conducted by seven trained Myanmar enumerators from November 2014 to January 2015. All the data were collected from farmers and no animal experiment or testing was involved in this study. The study was approved by the University of Queensland Human Research Ethics Committee (approval number #2014001425).

### Development of animal health and production measures that can been compared between different livestock ownership groups

We developed three indicators, a) ‘livestock health problems’, b) ‘biosecurity and livestock disease prevention index (BDPI)’ and c) ‘income generated from livestock sale’, to compare the health and production practices and their impacts across different livestock ownership groups. As diseases are a major constraint to livestock production [32–34], we considered overall measures of syndromic health status by body systems (i.e. physical disorder, respiratory disorders, digestive disorders, nervous disorders, skin, reproductive disorders) as an indicator for general livestock health and subclinical disease. Appropriate treatments, targeted vaccinations and improved biosecurity might help to reduce the impact of livestock diseases [25, 35], and we combined these interventions into a ‘biosecurity and disease prevention’ index as an indicator for preventive efforts made by farmers. Finally, as farm income generated is directly linked to the outputs of livestock production (i.e. milk, eggs) and the sales of animals. The estimation of income was conducted not taking account of production cost due to the lack of data [36, 37], we evaluated the income from livestock against other sources of household income [38, 39].

### Livestock health problems

The occurrence of clinical signs in each livestock species over the 12 months preceding the interview was summarized in the following body system categories (regardless of the age and sex of infected animal): physical problems (e.g. sore or abnormal hoof, foot or leg causing abnormal movement in ruminants; and twisted head and neck in chicken), respiratory disorders (e.g. coughing, sneezing, discharge from the nose or other breathing problems), digestive disorders (e.g. constipation or straining to defecate, or pain in the belly, diarrhoea), nervous disorders (e.g. blindness, circling, abnormal behaviour), skin disorders (e.g. loss of hair/wool/feather, abnormal colour or appearance of skin, such as scabs on surface), reproductive disorders (e.g. abortions, offspring born dead, discharge from vulva in ruminant and poor egg quality; abnormal shape of egg; soft egg shell in chicken), urinary disorders (e.g. difficulty / straining to urinate, abnormal urine colour in ruminant), sudden death (Please see questionnaire for details) [40, 41].

### Biosecurity and livestock disease prevention index (BDPI)

**Fig. 8:**
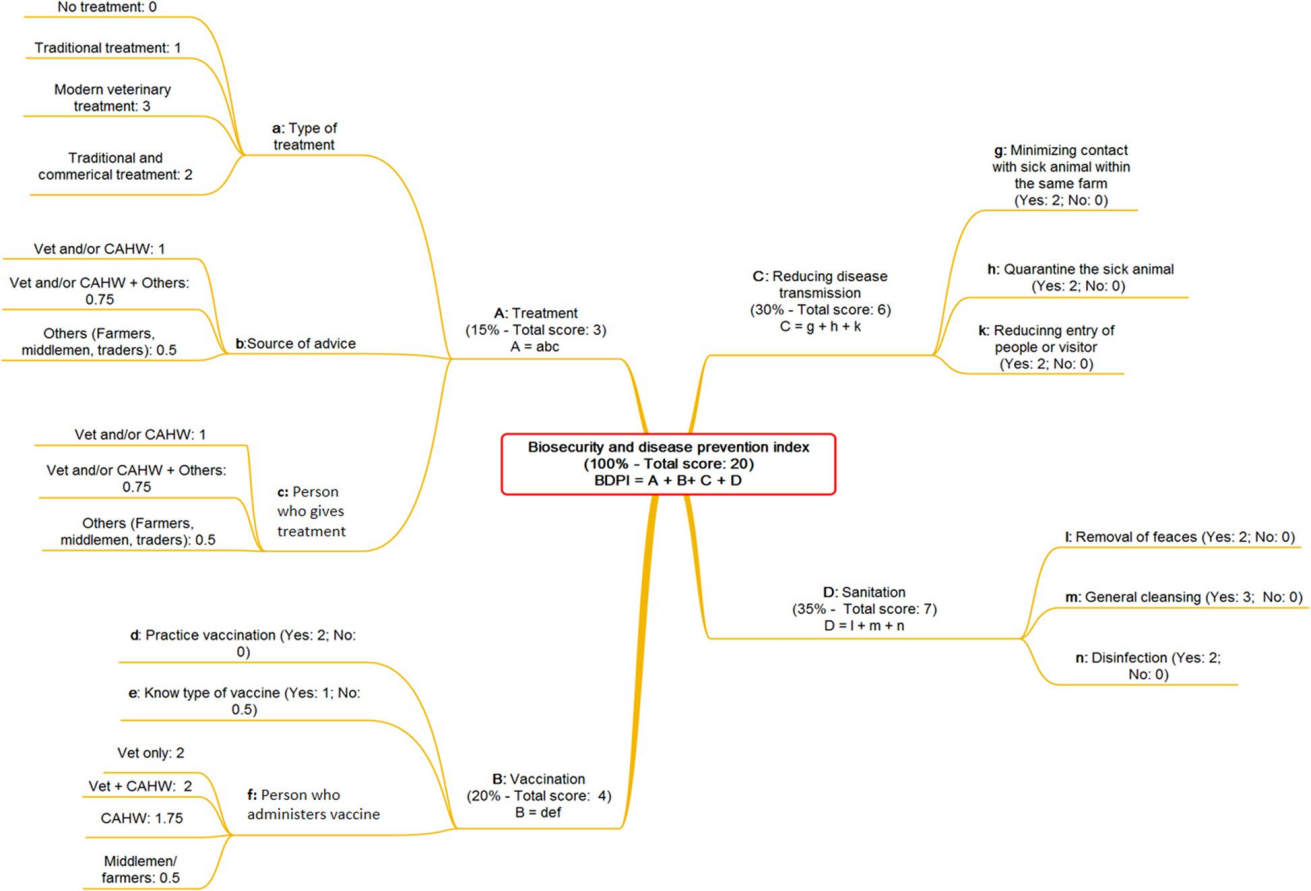
Flowchart for the calculation of the biosecurity and disease prevention index

Information of preventive health activities conducted by farmers was combined into a ‘biosecurity and livestock disease prevention index’ (BDPI). Information provided by farmers on four separate activities (treatment of livestock, vaccination of livestock, activities to reduce disease transmission and sanitation) were summarized in separate scores and then combined into a final weighted index measure (Fig. 8) [42].

Scores for treatment of livestock reflected the likely probability of success and were determined to indicate the skills and knowledge of the person(s) providing both the advice on treatment and its actual administration, and the treatment’s likely efficacy (i.e. a pharmaceutical product or a traditional remedy). The scores for vaccination of livestock reflected the likely probability of efficacy of the vaccination, based on whether or not it was conducted, the farmer’s awareness of the target disease or type of vaccine used, and the skills and knowledge of the person administering the vaccine. Scores for reducing disease transmission represented the sum of activities that would improve biosecurity and potential spread of infection between animals, in particular whether contact between sick and healthy animals was minimized on the same farm, how long a sick animal was segregated, and whether farm entry by other people was limited. Finally, scores for sanitation represented the sum of activities that would be likely to reduce indirect transmission of pathogens between animals: removal of faeces, general cleaning procedures on the farm (e.g. sweeping, cleansing the area with water and removing rubbish from the farm or surroundings) and disinfection practices.

Activities that contributed to treatment and vaccination of livestock were combined multiplicatively, whereas scores for activities to reduce disease transmission and improve sanitation were combined additively. Thus, activities under treatment and vaccination represented independent events, with probability of them happening together being the product of their individual score. For example, if treatment or vaccination of livestock was conducted, but by an inexperienced (lower scored) person, such as another farmer, the score for this action was proportionately reduced, compared to an experienced (higher scored) person, such as a veterinarian. On the other hand, scores for activities to reduce disease transmission represented a set of independent outcomes that in their union represented a stronger score. For example, implementation of quarantine of sick animals until recovery, minimizing contact with sick animals and reducing entry of people would result in the highest score, but fewer activities would result in lower scores.

We used weighting of the individual scores in the calculation of the overall index measure to represent how easily and how frequently activities were carried out by farmers, and how effective they were for various disease controls. Biosecurity practices such as activities to reduce disease transmission and sanitation were weighted with 30 and 35% respectively, while treatment of livestock and vaccination of livestock had weights of 15 and 20%. Weighting (15% treatment + 30% reducing disease transmission + 20% vaccination + 35% sanitation) and the scoring system to estimate the biosecurity and disease prevention practice (100%) was established was shown in Fig. 8. Thus, biosecurity practices accounted for a large proportion of the overall index (in particular for cattle farmers), while vaccinations had lower weightings (for example no vaccination was conducted by small ruminant farmers). This weighting also reflected that treatments or vaccinations alone would not provide excellent biosecurity on farms.

Factors that influence BDPI scores for each livestock species were then explored by percentile analysis. The highest limit of 50th percentile value was set as the highest limit number to categorise the results into three groups: no (Score 0), low (Score 1–45) and high (Score > 45) in cattle farms; no (Score 0), low (Score 1–12.5) and high (Score > 12.5) in small ruminant farms; and no (Score 0), low (Score 1–15) and high (Score > 15) in village chicken farms.

### Main income sources

To evaluate the importance of income from livestock sales in comparison to other income sources in the farming household, we established the scoring system using information provided by farmers during the interview as follows:

- Income generated from livestock sales per year
- Income from crop production per year
- Income from labour per year
- Income from trade per year
- Income received from relative per year

We then identified the top income source for each household and then summarized the frequency of the top income sources for each livestock ownership group.

### Statistical analysis

The data entry was conducted in a Microsoft Excel 2013 spreadsheet. Data were checked for data entry errors and validated by comparing digitized data with the original questionnaire by using NVivo Pro 11. Missing or suspicious data were discussed with interviewees over the phone. A causal diagram was created by using draw.io and NVivo Pro 11. Using Stata 14.0 (Stata Statistical Software, College Station, Stata Corporation, 2015), we used the survey-analysis approaches accounting for sampling weights, variance estimation (VCE), sampling strata (townships: primary sampling units PSUs) and clustering villages (secondary sampling units SSUs) [43–46].

Regression approaches were used for identifying associations between livestock management factors and livestock health problems and income from livestock sales considering hypothesized causal relationships. We used ordinal logistic regression for biosecurity and livestock disease prevention index (BDPI) and income from livestock sales, binomial logistic regression for presence-absence of livestock health problem for each body system. Thus, three regression models were developed for each livestock species (cattle, small ruminant, and village chicken). The proportional odds ratio assumption for ordinal regression models was tested by using the -omodel- command in STATA and the Brant test [47–49]. In addition, the variance of parallel regression analysis was tested by the significance test in the two tests [50]. Predictors significant at *p* < 0.05 in the univariable analyses were used firstly in the multivariable analysis, a forward selection and then backward elimination building procedure. The best fitted model was chosen by using Akaike Information Criterion (AIC).

## Supplementary Information

**Additional file 1.**

**Additional file 2.**

## Data Availability

The results for data that support the findings of this study are available in Tropical Animal Health and Production with the identifier: 10.1007/s11250-018-1738-9. The datasets used and/or analysed during the current study are available from the corresponding author on reasonable request. Supportive data and materials were also provided in supplementary materials. Table 1 Univariable analysis to identify factors affecting biosecurity and disease prevention indexes (BDPI) on cattle, small ruminant and village chicken farms in the CDZ of Myanmar. Table 2 Univariable analysis to understand factors affecting income generated from livestock sale cattle, small ruminant and village chicken farms in the CDZ of Myanmar.

## Abbreviations

CDZ: Central Dry Zone
UQ: The University of Queensland
FMD: Foot and Mouth Disease
ND: Newcastle Disease
ACIAR: Australian Center for International Agriculture Research
LBVD: Livestock Breeding and Veterinary Department
PSUs: Primary sampling units
SSUs: Secondary sampling units
VCE: Variance estimation
FPC: Finite population correction
mm: Millimetre
IQR: Interquartile rate
μ: Mean value
CI: Confident interval
HH: Household
AI: Artificial insemination
