# Supplementary material for: How can we compare multispecies livestock rearing households? – an analysis of the impact of health and production parameters on multispecies livestock rearing outcomes
Source: BMC Vet Res. 2022 Apr 29;18:158. doi: 10.1186/s12917-022-03175-x (PMC9052491; doi:10.1186/s12917-022-03175-x)
Supplement: Supplementary file 1 — Additional file 1. [file 12917_2022_3175_MOESM1_ESM.docx]

**Additional Materials**

Table 1 Univariable analysis to identify factors affecting biosecurity and disease prevention indexes (BDPI) on cattle, small ruminant and village chicken farms in the CDZ of Myanmar

| **Variables** | **Categories** | **N** | **BDPI (%)** | | | **Odds ratio** | **p-value** | **Wald test** |
| --- | --- | --- | --- | --- | --- | --- | --- | --- |
|  |  |  | **No** | **Low** | **High** |  |  |  |
| **Outcome variable: BDPI in cattle farm**  No (0%) – 20 (5.0%)  Low (1-45%) – 197 (51.1%)  High (>45%) – 165 (43.9%) | | | | | | | | |
| Duration of rearing cattle | <5 years | 382 | 22.1 | 11.9 | 4.7 | 1 |  | - |
|  | >5 years |  | 77.9 | 88.1 | 95.3 | 3.0 (1.0-9.0) | 0.049 |  |
| **Outcome variable: BDPI in small ruminant farm**  No (0%) – 79 (26.9%)  Low (1-12.5%) – 117 (36.8%)  High (>12.5%) – 107 (36.3%) | | | | | | | | |
| Duration of rearing sheep | <5 years | 303 | 77.9 | 86.5 | 94.7 | 1 |  | - |
|  | >5 years |  | 22.1 | 13.5 | 5.3 | 0.3 (0.2-0.6) | <0.0001 |  |
| **Outcome variable: BDPI in village chicken farm**  No (0%) – 126 (38.1%)  Low (1-15%) – 106 (32.9%)  High (>15%) – 95 (29.0%) | | | | | | | | |
| Type of animal reared | Village chicken only | 327 | 14.8 | 18.5 | 32.7 | 1 |  | 0.0026 |
|  | Cattle + Village chicken |  | 25.4 | 42.2 | 32.7 | 0.6 (0.2-1.8) | 0.391 |  |
|  | Small ruminant + Village chicken |  | 34.1 | 11.6 | 10.8 | 0.2 (0.1-0.5) | 0.001 |  |
|  | All 3 spp. |  | 25.7 | 27.8 | 23.7 | 0.5 (0.2-1.4) | 0.179 |  |
| Duration of rearing village chicken | <5 years | 327 | 31.2 | 22.4 | 16.0 | 1 |  | - |
|  | >5 years |  | 68.8 | 77.6 | 84.0 | 1.9 (1.2-2.9) | 0.004 |  |

Table 2 Univariable analysis to understand factors affecting income generated from livestock sale cattle, small ruminant and village chicken farms in the CDZ of Myanmar ********This cut-off represent the median income from the sale of animals of this livestock species*

| **Variables** | **Categories** | **N** | **Income (%)** | | | **OR** | **p-value** | **Wald test** |
| --- | --- | --- | --- | --- | --- | --- | --- | --- |
|  |  |  | **Low** | **Medium** | **High** |  |  |  |
| **Outcome variable: Income generated from livestock sale in cattle farmers**  No income (US$ 0) – 128 (36.9%)  Low (< US$ 450) – 127 (32.2%)*  High (> US$ 450) – 127 (30.9%) | | | | | | | | |
| Type of animal reared | Cattle only | 382 | 56.0 | 19.5 | 22.6 | 1 |  | <0.0001 |
|  | Cattle + Small ruminant |  | 13.2 | 9.7 | 22.5 | 4.1 (1.4-11.5) | 0.009 |  |
|  | Cattle + Village chicken |  | 20.6 | 40.8 | 25.9 | 3.0 (1.6-5.4) | 0.001 |  |
|  | Cattle + Small ruminant + Village chicken |  | 10.2 | 30.0 | 29.1 | 4.7 (2.4-9.3) | <0.0001 |  |
| Reproductive problem | No | 382 | 99.3 | 96.3 | 93.9 | 1 |  | - |
|  | Yes |  | 0.7 | 3.7 | 6.1 | 3.4 (1.3-8.9) | 0.012 |  |
| **Outcome variable: Income generated from livestock sale in small ruminant farms**  No income (US$ 0) – 55 (18.9%)  Low (< US$ 533) – 131 (39.9%)*  High (> US$ 533) – 117 (41.1%) | | | | | | | | |
| Digestive problem | No | 303 | 63.4 | 49.7 | 37.8 | 1 |  | - |
|  | Yes |  | 36.6 | 50.3 | 62.2 | 2.0 (1.3-3.2) | 0.003 |  |
| Reproductive problem | No | 303 | 75.9 | 63.8 | 45.0 | 1 |  | - |
|  | Yes |  | 24.1 | 36.2 | 55.0 | 2.6 (1.4-4.6) | 0.002 |  |
| **Outcome variable: Income generated from livestock sale in village chicken farms**  No income (US$ 0) – 72 (23.2%)  Low (< US$ 373) – 129 (39.7%)*  High (> US$ 373) – 126 (37.1%) | | | | | | | | |
| Types of animal reared | Village chicken only | 327 | 32.4 | 34.5 | 0 | 1 |  | <0.0001 |
|  | Cattle + Village chicken |  | 37.8 | 27.6 | 35.9 | 3.2 (1.8-5.5) | <0.0001 |  |
|  | Small ruminant + Village chicken |  | 11.1 | 14.5 | 31.2 | 7.5 (3.6-15.3) | <0.0001 |  |
|  | All 3 spp. |  | 18.7 | 23.4 | 32.8 | 4.8 (2.3-10.3) | <0.0001 |  |
