# Supplementary material for: How can we compare multispecies livestock rearing households? – an analysis of the impact of health and production parameters on multispecies livestock rearing outcomes
Source: BMC Vet Res. 2022 Apr 29;18:158. doi: 10.1186/s12917-022-03175-x (PMC9052491; doi:10.1186/s12917-022-03175-x)
Supplement: Supplementary file 2 — Additional file 2. [file 12917_2022_3175_MOESM2_ESM.pdf]

Township Survey on Observation of the Animal Production, Animal Health Care System, Trade and Marketing Network in the Central Dry Zone (CDZ) of Myanmar

Survey Objectives

- To observe and describe the traditional animal production system and current animal health care system currently practiced in the Central Dry Zone (CDZ) of Myanmar.
- To observe and describe farmer's attitude and awareness on animal diseases and major cross-species disease transmission in accordance with the one-health paradigm.
- To observe and describe the animal trade and marketing network in the Central Dry Zone of Myanmar
- To find out the most efficient, reliable and relevant solutions for the development of livestock production and one-health paradigm in the Central Dry Zone of Myanmar by analysing the observations from this survey

Declaration

According to the reports, it is found out that the livestock population is very high in the central part of Myanmar than other regions. Due to the reason of getting little rain in the central part of Myanmar, the central part of Myanmar become named as "Central Dry Zone of Myanmar", and people, who live in these areas, cannot rely on agriculture and crop production. As a consequence, the animal production become playing a critical role in the Central Dry Zone. Even though the animal production is popular in these areas, the farmers, practicing the animal production in these areas, have still faced with some dilemmas in their animal production such as animal management, animal diseases and trade. The information collected from the farmer will be confidential. The survey is conducted with the purpose of finding out the current animal production system, animal health problem in order to ensure that recommendation for production is useful for famers; and trade and marketing network in the Central Dry Zone of Myanmar, as part of my PhD study.

Information of Survey

1. Date (DD/MM/YY) ____
2. Name of interviewer ____

Location

3. GPS point ______________________________ 4. P-code __________________________

5. Region ______________________________
6. District ______________________________
7. Township ______________________________
8. Village tract ______________________________
9. Village ______________________________
10. Number of household _________________________

General Information of the Interviewee (Please tick √ the appropriate box)

11. Name - ______________________________ 12. Age - ( ) years old

13. Gender - Male ( ) Female ( )

14. Role of the interviewee in the household - (--)

15. Which of the following animal production do you have experience in?

(Please tick √ the appropriate box)

Species	No experience	<5 years	5-10 years	>10 years
Cattle				
Sheep				
Goat				
Village chicken				

16. Which of the following animal production do you rear today?

(Please tick √ the appropriate box)

Species	Rearing today
Cattle	
Sheep	
Goat	
Village chicken	
Other (Describe_____________________________)	

Household details

17. Number of family member (Permanent residents) ____________ people
18. Number of family member involving in animal production ____________ people

19. Do you hire labour for animal production? Yes ☐ No ☐

Home Asset Score (HAS) Of Small-Scale Animal Producers

20. Which of the following businesses contribute the largest amount of money to your household in a typical year?

(Please tick √ the appropriate box)

Agriculture	Livestock production	Labour	Shop or commercial business	Money from relatives not living in the household	Other (Please describe)

21. How much money did your household earn per month over the last 12 months?

Minimum	Average	Maximum

22. How many family members do you have in your household?

23. Which of the following are owned by your household?

(Please tick √ the appropriate box)

**Radio**	**TV**	**Bicycle**	**Motorbike**	**Fridge**	**Watch**	**Clock**	**Electric fan**	**Cart**	**Truck**	**Mobile phone**	**Landline phone**	**Sewing machine**	**Rice cooker**

24. Please describe your household's land and housing; and the land for your business? (Please tick √ the appropriate box)

**Own agricultural land**	**Rent agricultural land**	**Landless (Do not own and do not rent)**	**Shared house**	**House in owned land**	**House in rent land**	**House in shared land**	**Own extra land in this village**	**Own land in other villages**

25. Please describe the age and education level of household members.

**List**	**Gender**	**Family member and age**					**Did not attend school but:**			**Maximum education level**					
		**Total**	**< 10 y.o**	**≤ 18 y.o**	**18-40 y.o**	**> 40 y.o**	**illiterate**	**can read**	**can write**	**Primary School**	**Middle school**	**High school**	**Undergraduate**	**Graduate**	**Postgraduate**
Number of family members	Female														
	Male														

26. Please describe the materials used for your house.
(Please tick √ the appropriate box)

**Roof**									
None	Palm leaves	Thatch	Plastic sheet	Bamboo	Cement fiber	Brick	Metal	Wood	Other (Describe: _____________)

**Wall**											
None	Bamboo with mud	Stone with mud	Reused wood	Concrete wall	Wood	Bamboo	Palm leaves	Metal	Stone with lime	Brick wall	Other (Describe: _____________)

**Floor**							
Sand	Palm or Bamboo	Brick	Stone	Parquet	Mud/clay/ earth floor	Concrete	Other (Describe: _____________)

27. Please describe the eating style.
(Please write the number in the box)

**List**	**Number of days consumed**	**Out of how many days**
Meat		
Fish		
Vegetables		
Beans or peas		
Potatoes, etc.		

28. Please describe the material you use for the following.
(Please tick √ the appropriate box)

Cooking									
Mains electricity	Natural gas	Biogas	Kerosene	Coal	Wood	Straw or shrubs or grass	Agricultural crop	Dung	Other (Describe: _____________)

Lighting							
Mains electricity	Biogas	Kerosene	Candles	Fire wood	Battery	Solar light	Other (Describe: _____________)

29. Please describe the water and sanitation resources for your household.
(Please tick √ the appropriate box)

Drinking water												
Piped into dwelling	Pipe into yard or plot	Public tap or stand pipe	Open well in dwelling	Open well in yard or plot	Open public well	Protected well in dwelling	Protected well in yard or plot	Protected public well	River or lake	Dam	Water from bottle	Other (Describe : _____________)

Toilet							
Shared flush toilet to sewer	Own flush toilet to sewer	Own flush toilet to non-sewer	Use bush or field as latrine	Septic tanks or toilet	Pit as latrine	River or stream or creek	Other (Describe: _____________)

Cattle Production

Animal Production System

Type and Number of Animal

30. What kind of animal do you have in your farm? Please write down the number of each kind of animal owned by the household today and 12 months ago.

Time	Type of animal									
Today	Male calves:		Female calves:		Cows:		Bulls:		Castrated males:	
12 months ago	Male calves:		Female calves:		Cows:		Bulls:		Castrated males:	

Definition: Calves ≤ 12 months old; Cows > 12 months old (Female); Bull > 12 months old (Male)

31. What age do you usually castrate your animal

< 6 m.o ☐ 6-12 m.o ☐ > 12m.o ☐ Do not castrate ☐

(Please tick √ the appropriate box)

Housing

(Please tick √ the appropriate box)

32. Do you provide shelter for your animals? Yes ☐ No ☐

33. Please provide the specific information and materials used for the shelter. (Please tick √ the appropriate box)

Roof				Wall				Floor			
**None**	**Corrugate**	**Leave**	**Others**	**None**	**Bamboo**	**Wood**	**Others**	**None**	**Wood**	**Bamboo**	**Other**

34. Where do you provide their housing?

Underneath the house ☐ Extension of the house ☐ Separate building in the farm ☐ Away from the farm ☐ Other (describe: ______________) ☐

Feeding system

35. Indicate the importance of different kinds of feeding to the classes of cattle in your household by writing numbers in the table.

(Please write 1= a small amount or no feeding; 2= Moderate amount of the diet; 3= Most of all of the diet in the appropriate box)

**Season**	**Free grazing**	**Supplementary feedstuff**							**Class of animal**
		**Cut & carried Grass**	**Rice straw**	**Crop residues**	**Groundnut cake**	**Sesame cake**	**Sorghum**	**Other (Describe: ____________)**	
Summer									**All**
									**Bull & draught**
									**Cow**
									**Calf**
Rainy									**All**
									**Bull & draught**
									**Cow**
									**Calf**
Winter									**All**
									**Bull & draught**
									**Cow**
									**Calf**

36. What is the average <u>total</u> hours grazed per day? ______________________ hours

37. How many times are animals usually grazed each day? ______________________ times

38. What is the gender and age of the person(s) who does most supervision of free-grazing animals?

____

<u>Water supply</u>

39. What is the source of water provided at home?

(Please tick √ the appropriate boxes)

**River**	**Well**	**Pond**	**Tap water**	**Others (describe ___________)**	**Do not provide water at home**

40. How often do you usually provide water for your animals at home?

(Please tick √ the appropriate boxes)

Season	Type of animal	Daily	Several times per week	Once a week	Other (Describe)
Summer	Bull & draught				
	Cow				
	Calf				
Rainy	Bull & draught				
	Cow				
	Calf				
Winter	Bull & draught				
	Cow				
	Calf				

<u>Weaning Management</u>

41. At what age do you usually wean your calves?

≤ 3 m.o ☐ 4-6 m.o ☐ 6-12 m.o ☐ > 12 m.o ☐ Do not wean ☐

<u>Breeding Management</u>

42. Please specify the purpose of breeding.

To sell to others ☐ Replacement animal for my own herd ☐ Male- To hire for breeding ☐ Other (Describe: ___________) ☐ Do not breed ☐

43. If you breed, what is the source(s) do you use? Please tick √ the appropriate box(es)

Natural mating	
Own bull	
Another bull in village	
Bull from other village	
Other (Describe) ______________________	

Artificial insemination	
Township vet office	
Blue Cross Worker	
Private vet	
Other (Describe) ______________________	

Purpose of rearing and the role of importance in family’s income

44. Please describe the purpose and the role of importance in your family's income. Please tick √ the appropriate box(es)

**Meat**		**Milk**		**Draught power**		**Breeding**		**Others (Please fill the reasons)**	
Very important		Very important		Very important		Very important		Very important	
Important		Important		Important		Important		Important	
Not too important		Not too important		Not too important		Not too important		Not too important	

Definition:

Very important = Get more than 50% of the total income of a typical year
Important = Get more than 10-50% of the total income of a typical year
Not too important = Get less than 10% of the total income of a typical year

Cause of animal loss			Bull/draught			Cow			Calves (< 6 months)		
No.	Description	Clinical signs seen with the past two years	Frequency*	Number affected	Severity	Frequency*	Number affected	Severity	Frequency*	Number affected	Severity
45.	Physical Problem	Sore or abnormal hoof, foot or leg causing abnormal movement									
		Animal not growing as much as other animals from the household									
		Weakness, or frequently lying or sitting down									
46.	Respiratory Problem	Coughing , sneezing, discharge from the nose or other breathing problems									
47.	Digestive problems	Drooling or sores in the mouth									
		Unwillingness to eat or anorexia									
		Constipation or straining to defecate, or pain in the belly									
		Diarrhoea									
48.	Nervous System Problem	Examples: Blindness, circling, abnormal behaviour									

Cause of animal loss			Bull/draught			Cow			Calves (< 6 months)		
**No.**	**Description**	**Clinical signs seen with the past two years**	**Frequency***	**Number affected**	**Severity**	**Frequency***	**Number affected**	**Severity**	**Frequency***	**Number affected**	**Severity**
49.	Skin Problem	Itchiness/scratching									
		Loss of hair/wool, abnormal colour or appearance of skin, such as scabs on surface									
		Swelling or other problem with udder									
50.	Reproductive Problem	Abortions, Offspring born dead, discharge from vulva									
		Unable to mate or abnormal mating behaviour									
51.	Urinary System Problem	Difficulty / straining to urinate, abnormal urine colour									
52.	Sudden death	Found dead									
53.	Other Problems	Others (--)									

**Cause of animal loss**			**Bull/draught**			**Cow**			**Calves (< 6 months)**		
**No.**	**Description**	**Clinical signs seen with the past two years**	**Frequency***	**Number affected**	**Severity**	**Frequency***	**Number affected**	**Severity**	**Frequency***	**Number affected**	**Severity**
54.	Bad weather										
55.	Predators										
56.	Theft										

* Frequency: 0= Never; 1= Rarely; 2= Moderately frequently; 3= Very frequently or all the time

**Severity: 1= Not severe and recover; 2 = Severe but not death (recover); 3= Death

Treatment

57. What type of medical treatment you provide when your animal is sick?

- [ ] Traditional medicine
- [ ] Commercial medicine
- [ ] None

58. Who provides advice about managing health or treating illness of your cattle?

Person who give advices	Never	Very often	Often	Sometimes	Rarely
Veterinarians					
Middleman					
Neighbours					
Other farmers					
Relatives					
Yourself					
Blue Cross Worker					
Others (Describe________________________________)					

59. Who actually gives treatments or medicines to your cattle?

Person who give treatment	Never	Very often	Often	Sometimes	Rarely
Veterinarians					
Middleman					
Neighbours					
Other farmers					
Relatives					
Yourself					
Blue Cross Worker					
Others (Describe ______________)					

Animal Diseases Prevention and Control

Medical Based Prevention

60. Do you practice vaccination? ☐ Yes ☐ No

61. Do you know what type of vaccine you used? ☐ Yes ☐ No
62. If yes, please specify. (Describe ____)

63. How often do you practice vaccination for your animal?

Once a year	Twice a year	Three times a year	More than three times a year	Remarks

64. Who administer the vaccine to your animal?

Middleman	Blue cross worker	Vet	Relatives	Yourself	Others (Describe ___________)

Biosecurity Measure and Disinfection

65. If there is sick animal in your village, how do you usually prevent the disease transmission?

☐ Minimizing the contact of other animal to sick animal
☐ Quarantine the sick animal
☐ Reducing the entry of other people or visitors into the farm
☐ Disinfection the farm
☐ Others (Describe ___________________________________)

66. Which of the following do you usually do on your farm to help manage the health of your cattle?

☐ Removal of feces ☐ Sweeping ☐ Cleansing with water ☐ Disinfection ☐ Other (Describe______________)

Segregation

67. Do you usually segregate the sick animal? ☐ Yes ☐ No

68. Do you usually segregate the sick animal until it recover? ☐ Yes ☐ No

69. If yes, when it recovers, what do you do with it?

- ☐ Sell the animal
- ☐ Keep using for farm work
- ☐ Keep the animal together with other animals again
- ☐ Send it to other village (-----------------)
- ☐ Send it relatives' house (-----------------)
- ☐ Others ----------------------------------
- ☐ Do nothing

Sheep and Goat Production

Animal Production System

Type and Number of Animal

70. What kind of animal do you have in your farm? Please write down the number of each kind of animal owned by the household today and 12 months ago.

Time	Type of animal									
Today	Male lamb/kid:		Female lamb/kid:		Ewe/Dam:		Ram/Buck:		Castrated males:	
12 months ago	Male lamb/kid:		Female lamb/kid:		Ewe/Dam:		Ram/Buck:		Castrated males:	

Definition: Lamb/Kid≤ 12 months old; Ewe/Dam > 12 months old (Female); Ram/Buck > 12 months old (Male)

71. What age do you usually castrate your animal? < 6 m.o ☐ 6-12 m.o ☐ > 12m.o ☐ Do not castrate ☐

(Please tick √ the appropriate box)

Housing

(Please tick √ the appropriate box)

72. Do you provide shelter for your animals? Yes ☐ No ☐

73. Please provide the specific information and materials used for the shelter. (Please tick √ the appropriate box)

Roof				Wall				Floor			
**None**	**Corrugate**	**Leave**	**Others**	**None**	**Bamboo**	**Wood**	**Others**	**None**	**Wood**	**Bamboo**	**Other**

74. Where do you provide their housing?

Underneath the house ☐ Extension of the house ☐ Separate building in the farm ☐ Away from the farm ☐ Other (describe: _______________) ☐

Feeding system

75. Indicate the importance of different kinds of feeding to the classes of sheep or goat in your household by writing numbers in the table.

(Please write 1= a small amount or no feeding; 2= Moderate amount of the diet; 3= Most of all of the diet in the appropriate box)

Season	Free grazing	Supplementary feedstuff							Class of animal
		Cut & carried Grass	Rice straw	Crop residues	Groundnut cake	Sesame cake	Sorghum	Other (Describe: ____________)	
Summer									All
									Adult male
									Adult female
									Offspring
Rainy									All
									Adult male
									Adult female
									Offspring
Winter									All
									Adult male
									Adult female
									Offspring

76. What is the average total hours grazed per day? ______________________ hours

77. How many times are animals usually grazed each day? ______________________ times

78. What is the gender and age of the person(s) who does most supervision of free-grazing animals?

____

Water supply

79. What is the source of water provided at home?

(Please tick √ the appropriate boxes)

River	Well	Pond	Tap water	Others (describe ___________)	Do not provide water at home

80. How often do you usually provide water for your animals at home?

(Please tick √ the appropriate boxes)

Season	Type of animal	Daily	Several times per week	Once a week	Other (Describe)
Summer	Adult male				
	Adult female				
	Offspring				
Rainy	Adult male				
	Adult female				
	Offspring				
Winter	Adult male				
	Adult female				
	Offspring				

Weaning Management

81. At what age do you usually wean your lamb/ kid?

≤ 3 m.o ☐ 4-6 m.o ☐ 6-12 m.o ☐ > 12 m.o ☐ Do not wean ☐

Breeding Management

82. Please specify the purpose of breeding.

To sell to others ☐ Replacement animal for my own herd ☐ Male- To hire for breeding ☐ Other (Describe: ___________) ☐ Do not breed ☐

83. If you breed, what is the source(s) do you use?

Please tick √ the appropriate box(es)

**Natural mating**	
Own adult male	
Another male in village	
Male from other village	
Other (Describe) ______________________	

**Artificial insemination**	
Township vet office	
Blue Cross Worker	
Private vet	
Other (Describe) ______________________	

Purpose of rearing and the role of importance in family's income

84. Please describe the purpose and the role of importance in your family's income. Please tick √ the appropriate box(es)

**Meat**		**Milk**		**Breeding**		**Others (Please fill the reasons)**	
Very important		Very important		Very important		Very important	
Important		Important		Important		Important	
Not too important		Not too important		Not too important		Not too important	

Definition:

Very important = Get more than 50% of the total income of a typical year
Important = Get more than 10-50% of the total income of a typical year
Not too important = Get less than 10% of the total income of a typical year

Cause of animal loss			Adult male			Adult female			Offspring		
**No.**	**Description**	**Clinical signs seen with the past two years**	**Frequency***	**Number affected**	**Severity**	**Frequency***	**Number affected**	**Severity**	**Frequency***	**Number affected**	**Severity**
85.	Physical Problem	Sore or abnormal hoof, foot or leg causing abnormal movement									
		Animal not growing as much as other animals from the household									
		Weakness, or frequently lying or sitting down									
86.	Respiratory Problem	Coughing , sneezing, discharge from the nose or other breathing problems									
87.	Digestive problems	Drooling or sores in the mouth									
		Unwillingness to eat or anorexia									
		Constipation or straining to defecate, or pain in the belly									
		Diarrhoea									
88.	Nervous System Problem	Examples: Blindness, circling, abnormal behaviour									

Cause of animal loss			Adult male			Adult female			Offspring		
**No.**	**Description**	**Clinical signs seen with the past two years**	**Frequency***	**Number affected**	**Severity**	**Frequency***	**Number affected**	**Severity**	**Frequency***	**Number affected**	**Severity**
89.	Skin Problem	Itchiness/scratching									
		Loss of hair/wool, abnormal colour or appearance of skin, such as scabs on surface									
		Swelling or other problem with udder									
90.	Reproductive Problem	Abortions, Offspring born dead, discharge from vulva									
		Unable to mate or abnormal mating behaviour									
91.	Urinary System Problem	Difficulty / straining to urinate, abnormal urine colour									
92.	Sudden death	Found dead									
93.	Other Problems	Others (--)									
94.	Bad weather										

Cause of animal loss			Adult male			Adult female			Offspring		
**No.**	**Description**	**Clinical signs seen with the past two years**	**Frequency***	**Number affected**	**Severity**	**Frequency***	**Number affected**	**Severity**	**Frequency***	**Number affected**	**Severity**
95.	Predators										
96.	Theft										

* Frequency: 0= Never; 1= Rarely; 2= Moderately frequently; 3= Very frequently or all the time

**Severity: 1= Not severe and recover; 2 = Severe but not death (recover); 3= Death

Treatment

97. What type of medical treatment you provide when your animal is sick?

☐	Traditional medicine
☐	Commercial medicine
☐	None

98. Who provides advice about managing health or treating illness of your cattle?

Person who give advices	Never	Very often	Often	Sometimes	Rarely
Veterinarians					
Middleman					
Neighbours					
Other farmers					
Relatives					
Yourself					
Blue Cross Worker					
Others (Describe________________________________)					

99. Who actually gives treatments or medicines to your cattle?

Person who give treatment	Never	Very often	Often	Sometimes	Rarely
Veterinarians					
Middleman					
Neighbours					
Other farmers					
Relatives					
Yourself					
Blue Cross Worker					
Others (Describe ______________)					

Animal Diseases Prevention and Control

Medical Based Prevention

100. Do you practice vaccination? ☐ Yes ☐ No

101. Do you know what type of vaccine you used? ☐ Yes ☐ No

102. If yes, please specify. (Describe ____)

103. How often do you practice vaccination for your animal?

Once a year	Twice a year	Three times a year	More than three times a year	Remarks

104. Who administer the vaccine to your animal?

Middleman	Blue cross worker	Vet	Relatives	Yourself	Others (Describe ___________)

Biosecurity Measure and Disinfection

105. If there is sick animal in your village, how do you usually prevent the disease transmission?

☐ Minimizing the contact of other animal to sick animal
☐ Quarantine the sick animal
☐ Reducing the entry of other people or visitors into the farm
☐ Disinfection the farm
☐ Others (Describe ___________________________________)

106. Which of the following do you usually do on your farm to help manage the health of your cattle?

☐ Removal of feces ☐ Sweeping ☐ Cleansing with water ☐ Disinfection ☐ Other (Describe______________)

Segregation

107. Do you usually segregate the sick animal?

☐ Yes
☐ No

108. Do you usually segregate the sick animal until it recover?

- [ ] Yes
- [ ] No

109. If yes, when it recover, what do you do with it?

- [ ] Sell the animal
- [ ] Keep using for farm work
- [ ] Keep the animal together with other animals again
- [ ] Send it to other village (-----------------)
- [ ] Send it relatives' house (-----------------)
- [ ] Others ----------------------------------
- [ ] Do nothing

Village Chicken Production

Animal Production System

Type and Number of Animal

110. What kind of animal do you have in your farm? Please write down the number of each kind of animal owned by the household today and 12 months ago.

Time	Type of animal					
Today	Chick		Hen		Cock	
12 months ago	Chick		Hen		Cock	

Definition: Chick ≤ 22 months old; Hen >2 months old (Female); Cock >2 months old (Male)

Housing

(Please tick √ the appropriate box)

111. Do you provide shelter for your animals? Yes ☐ No ☐

112. Where do you provide their housing?

Underneath the house ☐ Extension of the house ☐ Separate building in the farm ☐ Bamboo coop ☐ On the tree ☐ Other (describe: ______________) ☐

Feeding system

113. Indicate the importance of different kinds of feeding to the classes of cattle in your household by writing numbers in the table.

(Please write 1= a small amount or no feeding; 2= Moderate amount of the diet; 3= Most of all of the diet in the appropriate box)

Season	Free grazing	Supplementary feedstuff						Class of animal
		Good quality rice	Broken rice	Rice bran	Broken pea	Household scrap	Others (Describe ___________)	
Summer								All
								Cock
								Hen
								Chick
Rainy								All
								Cock
								Hen
								Chick
Winter								All
								Cock
								Hen
								Chick

Water supply

114. What is the source of water provided at home?

(Please tick √ the appropriate boxes)

River	Well	Pond	Tap water	Others (describe ___________)	Do not provide water at home

115. How often do you usually provide water for your animals at home?

(Please tick √ the appropriate boxes)

Season	Type of animal	Daily	Several times per week	Once a week	Other (Describe)
Summer	Cock				
	Hen				
	Chick				
Rainy	Cock				
	Hen				
	Chick				
Winter	Cock				
	Hen				
	Chick				

Purpose of rearing and the role of importance in family's income

116. Please describe the purpose and the role of importance in your family's income. Please tick √ the appropriate box(es)

Meat		Egg		Breeding		Others (Please fill the reasons)	
Very important		Very important		Very important		Very important	
Important		Important		Important		Important	
Not too important		Not too important		Not too important		Not too important	

Definition:

Very important = Get more than 50% of the total income of a typical year
Important = Get more than 10-50% of the total income of a typical year
Not too important = Get less than 10% of the total income of a typical year

Cause of animal loss			Bull/draught			Cow			Calves (< 6 months)		
**No.**	**Description**	**Clinical signs seen with the past two years**	**Frequency***	**Number affected**	**Severity**	**Frequency***	**Number affected**	**Severity**	**Frequency***	**Number affected**	**Severity**
117.	Physical Problem	Twisted head and neck									
		Animal not growing as much as other animals from the household									
		Weakness, or frequently lying or sitting down									
118.	Respiratory Problem	Coughing , sneezing, discharge from the nose or other breathing problems									
119.	Digestive problems	Unwillingness to eat or anorexia; Constipation or straining to defecate; or pain in the belly									
		Diarrhoea									
120.	Nervous System Problem	Examples: Blindness, circling, abnormal behaviour									
121.	Skin Problem	Itchiness/scratching									
		Loss of feather, abnormal colour or appearance of skin, such as scabs on surface									

Cause of animal loss			Bull/draught			Cow			Calves (< 6 months)		
**No.**	**Description**	**Clinical signs seen with the past two years**	**Frequency***	**Number affected**	**Severity**	**Frequency***	**Number affected**	**Severity**	**Frequency***	**Number affected**	**Severity**
		Swelling or other problem with udder									
122.	Reproductive Problem	Poor egg quality; Abnormal shape of egg; Soften egg shell									
		Decreased egg production									
123.	Sudden death	Found dead									
124.	Other Problems	Others (--)									
125.	Bad weather										
126.	Predators										
127.	Theft										

* Frequency: 0= Never; 1= Rarely; 2= Moderately frequently; 3= Very frequently or all the time

**Severity: 1= Not severe and recover; 2 = Severe but not death (recover); 3= Death

Treatment

128. What type of medical treatment you provide when your animal is sick?

- [ ] Traditional medicine
- [ ] Commercial medicine
- [ ] None

129. Who provides advice about managing health or treating illness of your chicken?

Person who give advices	Never	Very often	Often	Sometimes	Rarely
Veterinarians					
Middleman					
Neighbours					
Other farmers					
Relatives					
Yourself					
Blue Cross Worker					
Others (Describe________________________________)					

130. Who actually gives treatments or medicines to your chicken?

Person who give treatment	Never	Very often	Often	Sometimes	Rarely
Veterinarians					
Middleman					
Neighbours					
Other farmers					
Relatives					
Yourself					
Blue Cross Worker					
Others (Describe ______________)					

Animal Diseases Prevention and Control

Medical Based Prevention

131. Do you practice vaccination? [ ] Yes [ ] No

132. Do you know what type of vaccine you used? ☐ Yes ☐ No

133. If yes, please specify. (Describe ____)

134. How often do you practice vaccination for your animal?

Once a year	Twice a year	Three times a year	More than three times a year	Remarks

135. Who administer the vaccine to your animal?

Middleman	Blue cross worker	Vet	Relatives	Yourself	Others (Describe ___________)

<u>Biosecurity Measure and Disinfection</u>

136. If there is sick animal in your village, how do you usually prevent the disease transmission?

- ☐ Minimizing the contact of other animal to sick animal
- ☐ Quarantine the sick animal
- ☐ Reducing the entry of other people or visitors into the farm
- ☐ Disinfection the farm
- ☐ Others (Describe ____________________________________)

137. Which of the following do you usually do on your farm to help manage the health of your cattle?

☐ Removal of feces ☐ Sweeping ☐ Cleansing with water ☐ Disinfection ☐ Other (Describe___ __________)

<u>Segregation</u>

138. Do you usually segregate the sick animal? ☐ Yes ☐ No

139. Do you usually segregate the sick animal until it recover?

- [ ] Yes
- [ ] No

140. If yes, when it recover, what do you do with it?

- [ ] Sell the animal
- [ ] Keep using for farm work
- [ ] Keep the animal together with other animals again
- [ ] Send it to other village (-----------------)
- [ ] Send it relatives' house (-----------------)
- [ ] Others ----------------------------------
- [ ] Do nothing
